# Oxidative Stress and Mitochondrial Dysfunction across Broad-Ranging Pathologies: Toward Mitochondria-Targeted Clinical Strategies

**DOI:** 10.1155/2014/541230

**Published:** 2014-05-04

**Authors:** Giovanni Pagano, Annarita Aiello Talamanca, Giuseppe Castello, Mario D. Cordero, Marco d'Ischia, Maria Nicola Gadaleta, Federico V. Pallardó, Sandra Petrović, Luca Tiano, Adriana Zatterale

**Affiliations:** ^1^Cancer Research Centre at Mercogliano (CROM), Istituto Nazionale Tumori Fondazione G. Pascale-IRCCS, 80131 Naples, Italy; ^2^Research Laboratory, Dental School, Sevilla University, 41009 Sevilla, Spain; ^3^Department of Chemical Sciences, Federico II University, 80126 Naples, Italy; ^4^National Research Council, Institute of Biomembranes and Bioenergetics, 70126 Bari, Italy; ^5^CIBERER, University of Valencia-INCLIVA, 46010 Valencia, Spain; ^6^“Vinca” Institute of Nuclear Sciences, University of Belgrade, 11070 Belgrade, Serbia; ^7^Department of Clinical and Dental Sciences, Polytechnical University of Marche, 60100 Ancona, Italy; ^8^Department of Genetics, ASL Napoli 1, 80136 Naples, Italy

## Abstract

Beyond the disorders recognized as mitochondrial diseases, abnormalities in function and/or ultrastructure of mitochondria have been reported in several unrelated pathologies. These encompass ageing, malformations, and a number of genetic or acquired diseases, as diabetes and cardiologic, haematologic, organ-specific (e.g., eye or liver), neurologic and psychiatric, autoimmune, and dermatologic disorders. The mechanistic grounds for mitochondrial dysfunction (MDF) along with the occurrence of oxidative stress (OS) have been investigated within the pathogenesis of individual disorders or in groups of interrelated disorders. We attempt to review broad-ranging pathologies that involve mitochondrial-specific deficiencies or rely on cytosol-derived prooxidant states or on autoimmune-induced mitochondrial damage. The established knowledge in these subjects warrants studies aimed at elucidating several open questions that are highlighted in the present review. The relevance of OS and MDF in different pathologies may establish the grounds for chemoprevention trials aimed at compensating OS/MDF by means of antioxidants and mitochondrial nutrients.

## 1. Introduction

Mitochondria have long been recognized as the main site of bioenergetic pathways [1, 2]. After the early investigations on OS in 1980s, implications for an involvement of mitochondria in OS were found in early 1990s [3, 4], which associated MDF with OS in the pathogenesis of some diseases, such as mitochondrial myopathies (progressive external ophthalmoplegia) and Parkinson's disease [5, 6]. Those early studies opened multiple research avenues toward growing and currently thriving investigations in a number of diseases, or disease groups, pertaining different medical disciplines. Beyond the focus on individual pathologies, or limited groups of diseases sharing molecular or clinical affinities, the present review is aimed at attempting a survey of OS/MDF across a broader range of different disorders that have been investigated for the occurrence of OS/MDF as pathogenetic mechanisms either directly or concomitant with other inborn or exogenous causes of disease.

## 2. Mitochondrial Diseases

A number of studies led to identifying a set of different disorders affecting mitochondrial function and/or structure that are collectively termed mitochondrial diseases (MDs) [7]. As shown in Table 1, primary mitochondrial diseases (PMDs) are caused by mitochondrial DNA (mtDNA) defects, while secondary mitochondrial diseases (SMDs) are caused by defects of nuclear genes encoding mitochondrial (or mitochondria-related) proteins [8]. In both cases, MDs have been associated with different deficiencies in mitochondrial functions and evidence has been reported for prooxidant states as clinical and/or molecular OS hallmarks [9–34]. Endogenous MDF in PMDs has been shown to affect oxidative phosphorylation (OXPHOS) activities in PMDs (Complex I, III, and/or IV), as in mitochondrial myopathy, encephalomyopathy, lactic acidosis, stroke-like symptoms (MELAS) [15–19], Kearns-Sayre syndrome [23], chronic progressive external ophthalmoplegia (CPEO) [24, 25], and Pearson syndrome [26, 27]. An overall OXPHOS inhibition has also been observed in SMDs, as Alpers-Huttenlocher syndrome, along with polymerase *γ* mutations [28–31]. Implications for OS in these disorders have been reported in terms of* COQ2* gene defect, encoding for OH-benzoate polyprenyltransferase catalyzing a major step in coenzyme Q10 synthesis. As a result, lower-than-normal CoQ10 levels were observed in mitochondrial neurogastrointestinal encephalomyopathy (MNGIE), a SMD and CoQ10 deficiency syndrome, a rare condition that causes MDF and includes a variety of clinical presentations as encephalomyopathy, ataxia, and renal failure [32, 33]. Protective effects of either CoQ10 or of other antioxidants were detected in Leber's hereditary optic neuropathy (LHON) [9–11], Leigh syndrome [12], neuropathy, ataxia, retinitis pigmentosa and ptosis (NARP) [14], MELAS [18, 19], and maternally inherited diabetes mellitus and deafness (MIDD) [22].

Altogether, MDs are recognized to involve the respiratory chain, both controlled by nuclear DNA and mtDNA. Mendelian mitochondrial defects can affect subunits of respiratory chain complexes, mitochondrial assembly proteins, mtDNA translation, phospholipid composition of the inner mitochondrial membrane, or mitochondrial dynamics or involve mtDNA maintenance, combining features of mendelian and mitochondrial genetics [34].

Beyond MDs, a broad and growing number of disorders have been investigated for the implications of OS in their respective pathogenetic mechanisms with concurrent involvement of MDF. Unlike strictly termed MDs, in most cases the available implications of MDF are indirect, relying on surrogate indicators, such as decreased levels of mitochondrial cofactors, or altered mitochondrial membrane potential (ΔΨ), or increased lactate/pyruvate ratio, or secondary mitochondrial damage as, for example, due to iron overload or to antimitochondrial autoantibodies. Even so, the range of MDF covers an extensive number of disorders that associate OS/MDF in their pathogenetic mechanisms, as reported previously for several disorders or groups of interrelated disorders and discussed below in the present review.

## 3. Genetic Diseases

A number of genetic diseases (GDs) have been investigated for the implications of OS/MDF in their pathogenesis, as summarized in Table 2 [35–101]. The broad range of GDs investigated in this context includes a set of cancer-prone and/or ageing-related disorders and a number of GDs affecting various tissues and organs, including CNS, blood, and muscles.

### 3.1. Cancer-Prone and/or Early Ageing Diseases

These GDs are characterized by an enhanced risk of malignancies that are often more prevalent in a given disorder (e.g., myeloid leukaemias in Fanconi anaemia, FA), and many of these GDs exhibit propension to early ageing, which either affects the whole organism (progerias, e.g., Werner syndrome, WS) or is confined to given tissues, such as bone marrow impairment or neurodegeneration. A huge body of literature, omitted in the present review, associates this disease group with deficiencies in DNA repair, and the majority of research efforts—and most of funding resources—have been deployed to investigate these GDs within the theorem of DNA repair deficiency, in view of planning gene therapy protocols.

In the case of Down syndrome (DS), the trisomic condition has been viewed beyond any effort of genetic engineering, and any therapeutic approach has been mostly confined to psychological and physical rehabilitation.

The involvement of OS in this disease group has been reported since early studies in 1980s finding redox abnormalities in cells from patients with DS [53], ataxia-telangiectasia (AT) [39], or FA [54]. As for mitochondrial abnormalities, a substantial body of literature has accrued for the implications of MDF in this group of diseases (see below); thus the occurrence of OS and MDF has been documented in cellular, molecular, and animal studies and by* in vivo* evidence from human patients.

#### 3.1.1. Ataxia-Telangiectasia

An established body of literature relates AT pathogenesis both with OS since 1983 [39] and with MDF. Evidence was provided for excess ROS production and oxidative DNA damage, and for mitochondrial abnormalities including ultrastructure aberrations, decreased ΔΨ and mitophagy, and increased expression of respiratory enzymes in AT cells [35–39].

#### 3.1.2. Bloom Syndrome

Early studies suggested the occurrence of OS in Bloom syndrome (BS) [40, 41]. We have previously reported excess oxidative DNA damage (8-hydroxy-2-deoxyguanosine, 8-OHdG) in WBC from BS patients, with an unexpected decrease in glutathione disulfide : glutathione (GSSG : GSH) ratio [42]. No reports were found to be evaluated regarding any MDF in BS phenotype, and this subject warrants* ad hoc* investigations.

#### 3.1.3. Cockayne Syndrome

Cockayne syndrome (CS) phenotype displays a set of OS hallmarks, such as excess reactive oxygen species (ROS) production and DNA oxidative damage, along with decreased 8-oxoguanine DNA glycosylase-1 (OGG1) expression [43, 44]. Mitochondrial abnormalities consisted of excess mitophagy [45], and Kamenisch and Berneburg found that defective CSA and CSB proteins localize to mitochondria, by decreasing interaction with mitochondrial OGG1 [46].

#### 3.1.4. Down Syndrome

Down syndrome (DS) represents one of the best documented GDs for the established implications of both OS and MDF in DS phenotype, both* in vitro* and* in vivo*, namely, human trisomy 21 patients and murine DS models, such as trisomy 16 [47–52]. Since the pioneering studies dating back to 1980s [53], DS phenotype has been associated with Cu,Zn-superoxide dismutase (SOD-1) overexpression. Thus the ratio SOD-1 to catalase (CAT) plus glutathione peroxidase (GPx) is increased; hence more hydrogen peroxide is generated by SOD-1 than CAT and GPx can catabolize, giving rise to an OS positive feedback. Valenti et al. [50, 51] investigated skin fibroblasts from DS patients, finding excess ROS production along with a deficiency in Complex I activity with the involvement of the cAMP/PKA signalling pathway [50]. This group also reported that epigallocatechin-3-gallate prevents OXPHOS deficit while promotes mitochondrial biogenesis in DS fibroblasts [51]. Other authors reported on MDF in DS, affecting Krebs cycle activities, CoQ10 deficiency and mitochondrial ultrastructure in human DS cells and in human patients [48, 49], and in trisomy 16 mice [52].

#### 3.1.5. Fanconi Anaemia

Fanconi anaemia (FA) has been associated with redox imbalances since early studies including, among others, the pioneering report by Joenje et al. [54]. Thereafter, a growing body of literature has established a number of mechanistic implications of OS in the functions of several proteins encoded by FA genes, as well as by* in vitro* and animal studies, and in blood cells and biological fluids from FA patients (reviewed in [55, 56]). Three independent studies of MDF in FA cell lines of different genetic subtypes provided evidence for abnormal ultrastructure and function of mitochondria in FA cells [57–59]. Our recent report on bone marrow cell transcripts from FA patients revealed downregulation of genes involved in mitochondrial functions, antioxidant activities, heat shock proteins, and chelating proteins [60]. Thus, the role in the FA phenotype of OS/MDF may be considered well established and awaits consequent interventions in clinical management, in view of preventing or delaying disease progression.

#### 3.1.6. Hutchinson-Gilford Syndrome

Hutchinson-Gilford syndrome (HGS) is a progeria displaying excess ROS production, and increased mRNA levels and protein content for mitochondrial Mn superoxide dismutase (SOD-2), along with a drop in the ATP (50%) content of HGS fibroblasts* versus* controls. Moreover, HGS cells showed defective caspase-like proteasome activity, and DNA damage was prevented by N-acetyl cysteine [61, 62].

#### 3.1.7. Nijmegen Breakage Syndrome

Nijmegen breakage syndrome (NBS) features oxidative DNA damage resulting in mitochondrial p53 accumulation and resistance to apoptosis, and in poly (ADP-ribose) polymerase (PARP) hyperactivation resulting in NAD^+^ depletion [63, 64]. Krenzlin et al. attributed the “extremely high incidence of malignancy among NBS patients to the combination of a primary double-strand break repair deficiency with secondary oxidative DNA damage” [64].

#### 3.1.8. Rothmund-Thomson Syndrome

Rothmund-Thomson syndrome (RTS) gene product RECQL4 localizes to the nucleolus in response to OS. RTS fibroblasts exhibit increased OS-related p38 MAP kinase; thus cell lifespan and growth rate are increased by p38 MAP kinase inhibitor [65, 66]. Moreover, RECQL4 was found to localize to mitochondria, and the loss of RECQL4 alters mitochondrial bioenergetics as mitochondrial reserve capacity was depressed after RECQL4 knockdown [67].

#### 3.1.9. Werner Syndrome

Werner syndrome (WS), also termed “adult progeria” due to its late phenotypic onset, has been extensively investigated for its relationships with OS [68–70], and we reported the multiple involvements of the defective WRN protein both in DNA stability and in redox balance [70]. Mitochondrial ultrastructure anomalies were found in cells from the WS mouse model, Wrn helicase mutant mice (Wrn^Dhel/Dhel^) [68, 71]. Alterations in OS endpoints were detected in blood cells and biological fluids from WS patients to the highest extent compared to patients with other OS-related diseases and* versus* healthy donors [72].

#### 3.1.10. Xeroderma Pigmentosum

Xeroderma pigmentosum (XP) displays several OS features, including defective repair of DNA oxidative damage (8,5′-(S)-cyclo-2′-deoxyadenosine), while lipid peroxidation and protein glycation are increased in cells and brains from XP patients, along with reduced expression of SOD [73]. Mitochondrial abnormalities were exhibited in terms of defective mitochondrial gene transcripts for 16 S rRNA, ATPase 6L, and lactate dehydrogenase; moreover, lower-than-normal CoQ10 levels were observed in serum from XP patients [74–77].

### 3.2. Neurological and Muscle Diseases

#### 3.2.1. Adrenoleukodystrophy

This genetic disease is characterized by progressive neurologic motor impairment and displays a set of OS-related defects. OS markers include decrease in GSH : GSSH ratio, excess lipid oxidation, and decrease in total antioxidant defenses in symptomatic but not in asymptomatic patients [78–80]. MDF was found to include impairment in OXPHOS activities and a decrease in Complex V, along with decreased ATP levels; moreover, mitochondrial inner membrane potential dissipation was reported [81]. A recent report found OXPHOS disruption and mitochondrial depletion in ABCD1 null mouse, a mouse model for adrenomyeloneuropathy [82].

#### 3.2.2. Duchenne Muscular Dystrophy

Duchenne muscular dystrophy (DMD) displays a set of OS hallmarks in terms of increased protein thiol oxidation, lipofuscin, 4-hydroxynonenal (4-HNE), and total hydroperoxides, with decreased GSH, in cells and biological fluids from DMD patients [83, 84]. A concomitant MDF was suggested by impaired OXPHOS activities and decrease in ATP synthesis in the mdx mouse model for DMD [85].

#### 3.2.3. Friedreich Ataxia

Friedreich ataxia displays a number of MDF features, including decreased Complex I, II, and III, aconitase, and CoQ10 levels, with mitochondrial Fe overload [86, 87]. A concomitant OS condition was shown by decreased vitamin E levels, Fe dysregulation, and hypersensitivity to OS [88].

#### 3.2.4. Huntington's Disease

Huntington's disease (HD) displays excess levels of brain protein carbonyls that are decreased by CoQ10 administration [89]. Moreover, CoQ10 and creatine exert additive neuroprotective effects in HD mice and rats. Evidence for MDF was provided by finding excess NADPH oxidase (NOX) activity in human HD brains, parallel with synaptosome fractions from cortex and striatum of HD (140Q/140Q) mice, suggesting that increased NOX2 activity at lipid rafts is an early and major source of OS and cell death in HD (140Q/140Q) neurons [89–92].

### 3.3. Other Genetic Diseases

#### 3.3.1. Hyperhomocysteinaemia

The set of dysmetabolic defects in hyperhomocysteinaemia (HHC) both include MDF and OS [93–95]. Evidence for MDF was provided by decreased ΔΨ, release of cytochrome-c and by increase in mitochondrial matrix metalloproteinase [93, 94]. Implications of OS in HHC phenotype were shown by a decrease in sulfhydryl groups and total antioxidant status in plasma from HHC patients [95].

#### 3.3.2. Sickle Cell Disease

Sickle cell disease (SCD) is mainly characterized by sickle erythrocyte haemolysis resulting in excess free Fe release that, as such, triggers OS. Thus, excess ROS production and advanced glycation end products (AGEs) were observed, with a concomitant GSH decrease [96, 97]. An early paper reported on Fe-laden mitochondria in sickled reticulocytes from SCD patients [98], and that early observation warrants further up-to-date investigations.

#### 3.3.3. Thalassaemia

Thalassaemic patients showed oxidative damage to lipid membranes, proteins, and DNA (WBC 8-OHdG) [99]. Mitochondrial damage was found to be induced by non-transferrin-bound Fe, resulting in decreased ΔΨ [100]. The observation of lower-than-normal levels of l-carnitine also might be related to MDF [101].

## 4. Ageing and Ageing-Related Degenerative Disorders

### 4.1. Ageing

Ageing is not regarded as a disease per se; however several diseases have been associated with ageing, as summarized in Table 3. Extensive and multi-decade-long literature relates ageing to OS and MDF. As a brief selection of this vast body of evidence, OS and nitrosative stress (NS) hallmarks were shown by excess age-related oxidative DNA damage, decreased GSH : GSSG ratio, increased inducible NOS (iNOS) expression, and Fe accumulation [102–104]. A major focus on age-related MDF has resulted in an established body of evidence including, among others, MDF related to decrease in both OXPHOS activities (Complexes I and IV) and Krebs cycle (downregulated pyruvate dehydrogenase along with overexpressed pyruvate kinase and lactate dehydrogenase). Moreover, Prdx3 overoxidization and mtDNA damage were reported [105–107].

### 4.2. Cardiovascular Diseases

Also cardiovascular diseases (CVDs) have been broadly investigated—since early studies—for the implications of OS/MDF in their pathogenesis. Double-edged physiopathological roles for ROS and reactive nitrogen species (RNS) are recognized, both as mediators of cardiovascular functions and as effectors of cardiovascular tissue damage [108, 109]. Among the best recognized OS/NS hallmarks, excess lipid and protein oxidation, altered NADPH regulation, and peroxynitrite formation have been reported [108, 109]. Well-established implications of MDF are available for CVDs, including reports on mitochondrial aberrations, SOD-2 upregulation, and mtDNA mutations in atherosclerotic plaques [110–113].

### 4.3. Metabolic Syndrome

Extensive evidence for OS/MDF implications in metabolic syndrome has been accumulating in recent years [114–119]. Evidence for OS-related changes was provided by excess TBARS and plasma 8-isoprostanes, along with decrease in total antioxidative activity and in serum vitamins C and E. An involvement for MDF in these disorders was found by downregulated Complex I, NADPH oxidase, and in SIRT3, leading to excess mitochondrial protein acetylation; also observed was a decreased mtDNA copy number and dysregulated carnitine palmitoyltransferase [114–119].

### 4.4. Osteoarthritis

Osteoarthritis (OA) features a number of OS hallmarks, as decreased total antioxidant capacity, thiol levels, catalase activity, and prolidase activity, along with increased total peroxides and lipid peroxides, and myeloperoxidase overexpression [120]. Evidence for MDF in OA patients was shown by downregulated SOD-2 and Complexes I, II, and III, and by mitochondrial genome dysregulation, with 17 upregulated and 9 downregulated genes [121–124].

### 4.5. Diabetes

Type 2 diabetes has been extensively investigated for its pathogenetic implications of OS/MDF. Diabetic patients displayed excess oxidation of proteins, lipids, and DNA, that is, increased levels of 15-F2t-iso-prostaglandin and 4-HNE, as products of lipid oxidation, and advanced oxidation protein products, advanced glycation end products (AGEs), excess oxidative DNA damage (8-OHdG), upregulated NADPH oxidase, Trx and HSP70, and decreased GSH : GSSG ratio [125–128]. Downregulation of Complex I and/or IV, and of SOD-2 point to MDF occurrence in diabetic patients [129–133]. It should be recalled that Type 2 diabetes occurs as a secondary clinical feature in some OS/MDF-related pathologies, such as the mitochondrial disease, MELAS [17], and genetic diseases, as Fanconi anaemia [55] and Werner syndrome [71].

## 5. Neurologic and Neuropsychiatric Diseases

The relevance and implications of MDF and OS has been investigated in a number of CNS-related diseases, as summarized Table 4. Disease-specific abnormalities in terms of OS/MDF are highlighted below.

### 5.1. Neurologic Diseases

#### 5.1.1. Alzheimer's Disease

Alzheimer's disease (AD) is characterized by *β*-amyloid deposition as a major neuropathological hallmark, which is related to a complement of OS-related alterations, such as oxidative damage to DNA, RNA, proteins, and lipids in synapses. Moreover, CoQ10 and MitoQ decrease OS endpoints and Fe metabolism in AD cells [134–136]. Concomitant MDF relates to OXPHOS alterations, that is, loss of Complex IV, and Complex V is oxidatively damaged and functionally altered. Mitochondria-associated ER membranes were found to be significantly increased in AD cells [134–139]. A recent paper reported on the 4-HNE-induced oxidation of lipoic acid that, in turn, affects lipoamide dehydrogenase, whose expression is significantly decreased in brains from human AD patients and from AD mice [138]. Zarrouk et al. [140] reported lipid alterations in AD patients that related to peroxisomal dysfunctions, via a cortical accumulation of saturated very long chain fatty acids (VLCFA), substrates for peroxisomal *β*-oxidation. This study investigated the effects at the mitochondrial level of VLCFAs that were tested on human neuronal SK-NB-E cells and found an inhibition of cell growth and mitochondrial dysfunctions that were observed by cell counting with trypan blue, MTT assay, and measurement of ΔΨ. VLCFA-treated-cells displayed stimulation of OS, along with lower levels of mitochondrial Complexes III and IV, changes of the cytoplasmic distribution of mitochondria, presence of large mitochondria, and enhancement of the mitochondrial mass [140]. The key involvement of peroxisomes in AD was reported by Kou et al. [141] in human postmortem brains; the patients were grouped into three cohorts of increasing severity (stages I-II, III-IV, and V-VI, resp.), based on the neuropathological Braak staging for AD on one hemisphere. Lipid analyses of cortical regions from the other hemisphere revealed accumulation of C22:0 and VLCFA, C24:0 and C26:0, all substrates for peroxisomal *β*-oxidation, in cases with stages V-VI pathology compared with those modestly affected (stages I-II). Confocal laser microscopy demonstrated a loss of peroxisomes in neuronal processes with abnormally phosphorylated tau protein, implicating impaired trafficking as the cause of altered peroxisomal distribution. These findings pointed to peroxisome-related alterations in AD, which may contribute to the progression of AD pathology [141].

#### 5.1.2. Amyotrophic Lateral Sclerosis

Amyotrophic lateral sclerosis (ALS) features a set of OS/MDF hallmarks. Familial ALS accounts for approximately 10% of cases and is associated with mutations of the gene encoding SOD-1 [142–144]. Abnormalities were found in endoplasmic reticulum proteins, with modifications of the Golgi network [143, 144]. Moreover, a decrease was reported in WBC glutathione peroxidase, SOD-1 and NADPH oxidase, and dysregulated Fe metabolism. MDF was detected in terms of decreased Complex I and ATP/ADP ratio and abnormal mitochondrial ultrastructure [144–146].

#### 5.1.3. Epilepsy

Epilepsy features NF*κ*B-induced upregulation of NOS II gene expression with NO-, ^∙^O_2_^−^-, and ONOO^−^-dependent decrease of Complex I activity and increased Complex-III-dependent ^∙^O_2_^−^ production of epileptic brain mitochondria; seizure-related ROS formation and a protective effect of acetyl-l-carnitine indicate concomitant OS in epilepsy [147–150]. Decrease of lipoic acid synthetase suggests inhibition of Krebs cycle along with defective mitochondrial energy metabolism [149].

#### 5.1.4. Myalgic Encephalomyelitis/Chronic Fatigue Syndrome

Myalgic encephalomyelitis/chronic fatigue syndrome (ME/CFS) both display OS and MDF hallmarks. Patients with ME/CFS showed excess urinary 8-OHdG, plasma lipid peroxides and serum oxidized LDL and decreased vitamin C, along with decreased vitamin E and HSP70. Mitochondria from ME/CFS patients displayed lower-than-normal CoQ10 levels, whereas ATP production was increased, and Complexes I, III, and IV activities were overexpressed [151–156].

#### 5.1.5. Multiple Sclerosis

Multiple sclerosis displays a number of OS hallmarks, such as increased WBC luminol-dependent chemiluminescence, carbonyl protein and MDA, nitric oxide metabolites, and total antioxidant capacity [157, 158]. Melatonin induced increased SOD and GPx and decreased MDA levels. The occurrence of MDF was shown by decreased OXPHOS activity and by mtDNA deletions and reduced PGC-1*α*, a transcriptional coactivator and master regulator of mitochondrial function [158–160].

#### 5.1.6. Parkinson's Disease

A pioneering report by Di Monte et al. in 1992 suggested a link between the observation of decreased GSH levels in Parkinson's disease and MDF [6]. More recent studies have found a number of altered OS-related endpoints, namely, excess plasma F2-isoprostanes, hydroxyeicosatetraenoic acid products, cholesterol oxidation products, neuroprostanes, phospholipase A2 and platelet activating factor-acetylhydrolase activities, urinary 8-OHdG, and dysregulated Fe metabolism [161, 162]. Evidence for MDF was provided by observations of decreased Complex V and CoQ10 levels, enhanced oxidation of cysteine residues within Complex I, and excess lactate [163–165].

### 5.2. Psychiatric Diseases

#### 5.2.1. Autistic Spectrum Disorders

Autistic spectrum disorders (ASD) include a group of paediatric and adolescent diseases that display a number of OS and MDF hallmarks. Extensive evidence for OS and NS in cells and biological fluids from ASD patients was reported by several independent studies, including decreased GSH : GSSG ratio in WBC and excess lipid peroxidation (4-HNE) in plasma and in RBC membranes [166–168]. Moreover, increased protein and DNA damage (3-nitrotyrosine, 3-NT, and 8-OHdG) was reported in frozen samples from necroptic cerebellum and temporal cortex of individuals with ASD [169]. The occurrence of both MDF in ASD patients includes downregulation of genes involved in electron transport chain (decreased Complex I, III, IV, and V) and in Krebs cycle (decreased aconitase expression), Mitochondrial DNA damage was reported, in terms of OS-mediated mtDNA deletions and transitions, namely, GC→AT and GC→TA [170–172]. Altogether, the established evidence for OS/NS/MDF in ASD patients sets realistic grounds for clinical interventions aimed at counteracting these metabolic imbalances in ASD patients.

#### 5.2.2. Bipolar Disorder

Evidence for OS in bipolar disorder (BD) has been provided in terms of lipid peroxidation and reduced Na^+^-K^+^-ATPase activity, which can be counteracted by lithium treatment [173]. Moreover, decreased plasma levels of total glutathione and GSH, together with lower catalase expression, increased protein carbonyls, 4-HNE, and 3-NT were found in BD patients [174, 175]. Mitochondrial abnormalities in BD patients displayed decreased attachment of hexokinase 1 to outer mitochondrial membrane and decreased Complex I levels [175].

#### 5.2.3. Major Depression

Major depression (MD) has been reported to be associated with OS and with inflammation endpoints, namely, excess levels of IL-6, IL-10, and IL-6/IL-10 ratio* versus* F2-isoprostanes, along with increased protein carbonylation and expression of GR, GPx, SOD, and CAT [176–178]. A study of postmortem prefrontal and parieto-occipital cortices of MD patients revealed downregulated mRNA and protein levels of Complex I subunits (NDUFV1, NDUFV2, and NADUFS1) [179].

#### 5.2.4. Obsessive-Compulsive Disorder

Obsessive-compulsive disorder (OCD) features some OS hallmarks, as excess MDA/TBARS and SOD-1, along with decreased GSH : GSSG ratio [180, 181]. A study reported on a shift in mitochondrial SOD-2 genetic subtype, with increased prevalence of the TBARS-related CC (Ala/Ala) genotype* versus* CT (Ala/Val) genotype in OCD patients that was significantly lower than in healthy donors [182].

#### 5.2.5. Schizophrenia

An extensive body of evidence points to the occurrence of OS, NS, and proinflammatory condition in schizophrenia (SZ) [183]. In particular excess lipid peroxidation, damage to proteins and DNA, decreased plasma total antioxidant status, and antioxidant levels (Trx, CoQ10, Vit. E, GSH, and melatonin) were observed in SZ patients, along with autoimmune responses, as excess IL-6 and PCC levels [184–186]. An involvement of MDF in SZ pathogenesis is shown by a recent report on a significant decrease in Complex I activity both* versus* health donors and* versus* BD patients [186] and suggested by the abovementioned decrease in CoQ10 levels [187].

## 6. Cancer

A huge body of literature—of over 12,000 MedLine citations—associates malignancies of several organs and tissues with OS/MDF. While recognizing the outstanding differences in the pathogenesis of different malignancies; however general remarks to the involvement of OS/MDF in carcinogenesis may be provided, as reviewed recently [188, 189]. An OS condition produces irregular cell membrane borders as a result of lipid peroxidation, breaks, and considerable DNA damage, resulting in mutations and eventually cancer, via oncogene activation or tumour suppressor gene inactivation. These mutations may initiate carcinogenesis. Once initiated cellular transformation, cancer cells display an increased anaerobic glycolysis enabling to support neoplastic proliferation (Warburg effect) [190]. During cancer promotion, hypoxic condition prevents pyruvate, produced by anaerobic glycolysis, to be converted into Acetyl-CoA and to enter into Krebs cycle (MDF). The accumulated pyruvate is disposed through an alternative pathway which leads to the formation of lactate, which decreases microenvironmental pH. In this phase, therefore, glycolysis is not affected by hypoxia and continues producing pyruvate and reaction intermediates useful to the pentose phosphate pathway that lead to the ultimate synthesis of purine and pyrimidine bases, essential to cellular high replicative activity. Thus, Krebs cycle is affected by hypoxia and stops. During cancer progression, acid microenvironment promotes tumour angiogenesis. The restored oxygen supply activates aerobic glycolysis but the cells continue to produce lactic acid even in presence of oxygen and MDF endures. Further microenvironmental acidification was related to the metastatic phenomenon [189].

Brief examples of some selected malignancies will be cited herein, as shown in Table 5. Though relevant, literature on the involvement of OS/MDF in preneoplastic lesions (e.g., leukoplakia) or cancer-predisposing diseases (e.g., hepatitis C) is not cited here.

### 6.1. Bladder Cancer

Evidence for OS and NS hallmarks in bladder cancer was provided by excess oxidative DNA damage (8-OHdG), 3-NT, and by peroxiredoxin 4 (Prdx4) upregulation that were associated with poor prognosis [191, 192]. An involvement of MDF in bladder cancer was reported by finding excess mtDNA mutations (G8697A, G14905A, C15452A, and A15607G) [193] and altered mitochondrial expression profile of proteins involved in OXPHOS, glycolysis/gluconeogenesis, and Krebs cycle [194].

### 6.2. Breast Cancer

Extensive studies have focused on the involvement of OS/MDF in breast cancer pathogenesis [195–197]. Among OS endpoints, excess 8-OHdG and protein carbonyl were reported, along with upregulated total SOD, SOD-1, and EC-SOD in plasma and breast tumours. Moreover, BRCA1 mutations were found to cause OS in tumour microenvironment [198, 199]. Implications for MDF in breast cancer include upregulation of >95 gene transcripts associated with mitochondrial biogenesis and/or translation. Moreover, phosphorylated BRCA1 was found to locate to mitochondria, and disease progression-related decreased CoQ10 levels and SOD-2 expression were reported [199–201].

### 6.3. Cervical Cancer

Cervical cancer features excess lipid peroxidation, with overexpressed GPx and decreased SOD and CAT expression [202–206]. Null mutations in GSTM1 and GSTT1 polymorphisms were associated with excess risk of cervical neoplasia [207]. Mitochondrial Prdx3 was found to be overexpressed in high-risk patients [208].

### 6.4. Colorectal Cancer

Colorectal cancer (CrC) is characterized by excess oxidative DNA damage (8-OHdG) that was correlated to CD80 mRNA mucosal levels; SOD, GR, and GPx overexpression was observed, with GST inhibition [209, 210]. The relative mtDNA copy number showed a U-shaped association* versus* CrC risk [211].

### 6.5. Endometrial Cancer

Endometrial cancer (EC) features OS-related anomalies by decreased SOD and CAT expression, along with increased lipid hydroperoxides and glutathione reductase (GR) [212]. In type I EC the occurrence of pathogenic tumour specific mtDNA mutations as well as a reduction of assembled Complex I in tumour samples were found associated with an activation of antioxidant enzymes and with an increase of mitochondrial biogenesis. Tissue from EC showed doubling citrate synthase activity, the mitochondrial DNA content, and the TFAM level [213, 214].

### 6.6. Gastric Cancer

Gastric cancer features excess 8-OHdG levels and decreased expression of 8-oxoguanine glycosylase (OGG-1) [215, 216]. An involvement of MDF in gastric cancer was suggested by SOD-2 overexpression and by the recent report that found* H. pylori*-induced mtDNA mutations and a decrease of mtDNA content [215, 217].

### 6.7. Hepatocellular Carcinoma

Hepatocellular carcinoma (HCC) has been investigated extensively for its pathogenetic implications of OS/MDF, also related to other nonneoplastic or preneoplastic liver disorders [218–221]. Among OS-related hallmarks, HCC was found to display excess 8-OHdG and 4-HNE associated with microvessel density, vascular endothelial growth factor, and Akt activity; moreover, d-ROM, *α*-fetoprotein, and fasting plasma were associated with HCC recurrence [218, 219]. Implications of MDF were reported by the association of mitochondrial SOD-2 and Trx* versus* patients' survival rate and by excess mtDNA 9-bp deletion polymorphism in HCC patients [220, 221].

### 6.8. Lung Cancer

A number of studies provided extensive evidence for the involvement of OS and MDF in lung cancer (LC) [222–228]. The altered OS endpoint found in LC included excess aldehyde levels, both in plasma MDA and in exhaled breath, and with decreased serum antioxidant capacity. Oxidative DNA damage was both reported as excess levels of 8-OHdG and of thymidine glycol in ever-smokers. A recent meta-analysis confirmed a strong association between a variant OGG1 Ser326Cys and the risk of developing lung cancer, stressing the role of oxidative DNA damage repair in this clinical setting [223, 227]. Moreover, haeme oxygenase-1 (HO-1) was found overexpressed, while CAT and carbonic anhydrase were downregulated [223–225]. An involvement of MDF in LC was associated with decrease in mtDNA and lower-than-normal CoQ10 levels; moreover, mt HSP90s was associated with worse clinical outcome; mitochondria were found to display increased ΔΨ [226–228].

### 6.9. Melanoma

An increased oxidative DNA damage (8-OHdG) was found in melanoma patients' cells that was associated with a poor prognosis, with increased NOS1 polymorphism (rs2682826) and increased TNF-*α* levels enhancing the action of tumour-associated macrophages [229–231]. Mitochondria from metastatic melanoma cells displayed increased expression of OXPHOS activities* versus* nonneoplastic melanocytes [232].

### 6.10. Myeloid Leukaemias

Blood cell malignancies have been extensively investigated for the pathogenetic implications of OS/MDF; however most of this literature appears to be focused on myeloid leukaemias (ML); thus the literature in this subject regarding lymphoid leukaemias is omitted. A number of studies have provided substantial evidence for OS hallmarks in ML, including excess lipid peroxides (measured as MDA, TBARS, and LOOH) and protein carbonyls and decreased thiol plasma levels and ferric reducing ability of plasma (FRAP); moreover, increased expression of CAT, Trx, adenosine deaminase, and xanthine oxidase, along with decreased Prdx4 were reported, and GSTP1 Ile105Val polymorphism was associated with ML [233–238]. Anomalies in mitochondrial structure and function in ML included a disease-related 2- to 50-fold increased amplification of mtDNA, survival advantage in patients with SOD-2 T* versus* C polymorphism (in codon 16 of the mitochondrial targeting sequence of SOD-2), and a decrease in mitochondrial-encoded genes against an increased mtDNA copy number [239–241].

### 6.11. Oral Cancer

Oral cancer displays both OS and NS hallmarks, with excess MDA and NO levels, and AGEs levels. Decreased CAT and SOD expression was found in OC tissue, while SOD expression was enhanced in erythrocytes [242–245]. As an indicator of MDF, somatic mutation of D-loop of mtDNA was associated with better survival [246].

### 6.12. Thyroid Oncocytic Carcinoma

Thyroid oncocytic carcinoma (TOC) is characterized by mitochondrial hyperplasia and harbours high loads of disruptive mtDNA mutations, the large majority in respiratory Complex I genes. The analysis of a TOC cell line (XTC.UC1) displayed a complete loss of Complex I, a reduced rate of respiration and ATP synthesis driven by Complex I substrates, and an increase of ROS production [247, 248].

## 7. Autoimmune Diseases

This class of diseases (Table 6) is characterized by a number of OS-related hallmarks; as for MDF, most of abnormalities may be ascribed to antimitochondrial autoantibodies (AMA). Thus, most of the observed MDF endpoints are secondary to AMA-induced damage.

### 7.1. Antiphospholipid Syndrome

Antiphospholipid syndrome (APS) features a set of OS- and inflammation-related altered endpoints, including excess serum amyloid A, C-reactive protein, 8-isoprostane and prostaglandin E2, increased peroxide production, nuclear abundance of Nrf2, and decreased antioxidant enzymatic activity and intracellular GSH [249]. Mitochondria from APS patient cells displayed altered ΔΨ; moreover, beneficial effects of CoQ10 supplementation were observed, by improving the preservation of mitochondrial ultrastructure [250].

### 7.2. Pemphigus Vulgaris

Pemphigus vulgaris displays OS-related alterations including excess lipid peroxidation (MDA), decreased antioxidant levels (Vit. A and E), and altered expression of CAT and GPx [251–253]. Autoantibodies against mitochondrial, desmosomal, and other keratinocyte self-antigens were reported [253, 254].

### 7.3. Primary Biliary Cirrhosis

Patients with primary biliary cirrhosis (PBC) display decreased levels of antioxidant vitamins (A, C and E), excess HOCl release associated with eosinophil peroxidase, and decreased GST expression [255–257]. The autoimmune condition in PBC was found to show IgG production against lipid peroxides and serum and saliva AMA against E2 subunits of 2-oxoacid dehydrogenase enzymes (pyruvate dehydrogenase complex, 2-oxo-acid dehydrogenase, and 2-oxo-glutarate dehydrogenase) pointing to autoimmune onset of MDF [258].

### 7.4. Psoriasis

This disorder features a number of OS- and NS-related hallmarks including excess lipid peroxidation (MDA and LOOH), oxidative DNA damage (8-OHdG), and nitrogen oxides (NOx) levels, with decreased total antioxidant status and GSH levels. Decreased expression of CAT, SOD, and GPx was observed [259–261]. Moreover, mitochondrial damage could be inferred by release of mitochondrial cytochrome c and by upregulated CYP4F8 mRNA [262, 263].

### 7.5. Rheumatoid Arthritis

Rheumatoid arthritis was found to display excess lipid peroxide levels, along with increased expression of myeloperoxidase and excess ROS production in neutrophils from peripheral blood and synovial infiltrate. This finding was related to downregulation of CAT, SOD, and GPx and decreased GSH levels, along with TNF-*α*-associated ER stress. Immune imbalance included excess AGE-IgG and chemokine (MCP-1/CCL2, RANTES/CCL5)-associated ROS [264–268]. Mitochondrial damage was reported as mtDNA mutations that were positively correlated with macroscopic synovitis [269].

### 7.6. Sjøgren's Syndrome

A number of OS- and NS-related hallmarks characterize Sjøgren's syndrome (SS) patients, including excess protein carbonyls and AGEs, 4-HNE, TNF-*α*, MPO, and 3-NT; moreover, excess 8-OHdG and hexanoyl-lysine were found in SS patients. Excess expression of Trx, LDH and mitochondrial GOT were found in these patients [270–273]. An autoimmune-based MDF was reported by finding antimitochondrial autoantibodies in SS patients' saliva [274].

### 7.7. Systemic Lupus Erythematosus

Systemic lupus erythematosus patients display a set of OS hallmarks, such as decreased CAT, SOD, and GPx expression, with decreased GSH levels, and excess chemokine-associated ROS production [275, 276]. Defective mitochondrial function was related to lower-than-normal ATP levels, increased ΔΨ, and persistent mitochondrial hyperpolarization [264, 276].

### 7.8. Systemic Sclerosis

Patients with systemic sclerosis (SSc) display some altered OS endpoints that include excess MDA, dityrosines, and carbonyls, with lower-than-normal uric acid levels, GSH, and total antioxidants status in saliva. Decreased SOD expression was accompanied by increased GPx expression [277–279], and mitochondrial damage was associated with high-sensitivity C-reactive protein and AMA in SSc patients [280].

### 7.9. Vitiligo

Vitiligo patients feature a set of OS-related hallmarks, such as excess 4-HNE with decreased cardiolipin and GSH levels. A decreased expression of MPO, GST, and CAT was reported, and GST polymorphism displayed excess GSTM1 null and GSTT1/GSTM1 double-null type [281, 282]. An involvement of MDF in vitiligo was suggested by altered OXPHOS lipid-dependent subunits [283].

## 8. Miscellaneous Pathologies

Two disorders and a pathologic condition that could not be ascribed to the above disease classifications are discussed for their pathogenetic implications of OS/MDF in Table 7.

### 8.1. Cataract

Patents with cataract display excess lipid peroxidation (TBARS) with decreased thiol levels and ferric reducing/antioxidant power; moreover, an increase in dehydroascorbate/ascorbate ratio was observed [284, 285]. A study of polymorphisms of SOD-1, CAT, and GPx genes in cataract patients found that G/G genotype of the SOD-1 (251A/G polymorphism) was associated with an increased risk of cataract [286]. Several reviews discuss the roles of mitochondria in cataract pathogenesis [287], and a H_2_O_2_-specific induction of mitochondrial Prdx3 in the eye lens was observed [288].

### 8.2. Fibromyalgia

Fibromyalgia (FM) is characterized by excess lipid peroxidation and TNF-*α* levels, with loss of total antioxidant capacity [289–291]. A line of studies by Cordero and his colleagues has focused on MDF in FM, reporting on decreased ΔΨ and CoQ10 levels, along with excess mt ^∙^O_2_^−^ production and excess mitophagy [291–293].

### 8.3. Malformations

When mechanistic aetiology is available, malformations may occur as a result of exposures to xenobiotics before or during pregnancy (reviewed in [294]), or some organ-specific malformations may be ascribed to endogenous mechanisms in the phenotype of some genetic diseases, as for example, Fanconi anaemia (reviewed in [55]). Whether teratogenic outcomes derive from exogenous or endogenous factors, a vast body of literature has focused on OS- and MDF-related pathogenetic mechanisms. The induction of developmental defects and malformations has been associated with OS and MDF in teratogenic action [295]. Thalidomide was reported to cause embryonic DNA oxidation in susceptible yet not in resistant species, which was associated with GSH depletion, or inhibition of GPx or GR, and with decreased mitochondrial NADH oxidase in teratogen-sensitive embryonic tissues [296, 297]. Ethanol was reported to upregulate NOX regulatory subunits and increases NOX expression [298]. An involvement of MDF in developmental anomalies was suggested by alterations of mitochondrial respiration [299] and by reduced activities of respiratory chain Complexes I and IV and ATP synthase in a murine model of fetal alcohol syndrome [300].

## 9. Discussion

“Free radical theories” have been proposed for ageing since 1956 by Harman [301, 302] and subsequently for some high impact disorders as cardiovascular diseases [303], diabetes [304], and neurodegenerative diseases [6, 305–307]. Almost sixty years after Harman's hypothesis, many individual disorders—or groups of interrelated disorders—have been investigated for the implications of OS in their respective pathogenetic mechanisms. An important step in this research line was provided by the recognition, in 1990s, of the direct association of MDF with OS [3–5].

In spite of the outstanding information accumulated in these decades, one may notice that the database is usually confined to each medical discipline and, to the best of our knowledge, a comprehensive and interdisciplinary view is still missing or partial. Moreover, the clinical management of several OS/MDF-related disorders often disregards this information, by failing to adopt therapeutic or chemopreventive means targeted to compensate OS/MDF.

Among the OS-related endpoints, the most commonly observed abnormalities across broad-ranging disorders were found by means of (a) oxidation products of biomolecules, including lipid peroxidation products, DNA hydroxyl adducts, advanced glycation end-products, and protein carbonyls; (b) abnormal expression or regulation of antioxidant activities, namely, SOD, CAT and glutathione-related activities (GR, GPx, GST), (c) glutathione levels and GSH:GSSG ratio, and (d) defects in iron metabolism.

Definitely less investigated than OS, the NS-related endpoints, as 3-nitrotyrosine and NO bioactivity, have been reported to be associated with other redox-related anomalies in some diseases, such as diabetes [127, 128], bladder cancer [191], autism [169], schizophrenia [183], and Sjøgren's syndrome [271]. Albeit relatively unexplored, the subject of nitrosative damage deserves higher attention in further studies, as NS-related endpoints might provide precious insights in the pathogenesis of several OS/MDF-related disorders.

Almost invariably, MDF hallmarks were detected in OS-related disorders in the evaluated literature, thus providing an overall pattern of an OS/MDF association, as expected on the grounds of a MDF-related onset of OS [3]. The most frequently observed changes in MDF, and in mitochondrial ultrastructure, included (a) changes in one or more activities of the electron transport chain, OXPHOS, that is, Complexes I to V; (b) decreased expression of Krebs cycle-associated activities; (c) defects in mitochondrial SOD-2 and/or Prdx3 expression; (d) mtDNA deletions, decreased mtDNA copy numbers and mitophagy [307]; (e) decreased levels of CoQ10 and/or of ATP; (f) changes in lactate/pyruvate ratio; and (g) changes in mitochondrial membrane potential (ΔΨ). Alterations in OXPHOS activities were most commonly reported across a broad range of disorders displaying MDF hallmarks. Relatively few studies reported on changes in Krebs cycle-related activities, such as pyruvate dehydrogenase and pyruvate kinase in ageing [105], lipoamide dehydrogenase in Alzheimer's disease [138], lipoic acid synthetase in epilepsy [149], and aconitase in Friedreich ataxia [87]. Another relevant biomarker of MDF, SOD-2 expression, was reported to be increased in gastric cancer [215] and hepatocellular carcinoma [220]; SOD-2 activity was found increased in atherosclerosis [111], type 2 diabetes mellitus [131], and upregulated SOD-2 transcripts in Hutchinson-Gilford syndrome [61]. Altogether, the observed overexpression or upregulation of SOD-2 may suggest a homeostatic response in mitochondrial redox balance. The mitochondrial peroxidases, Prdx3 and Prdx4, were found to display expression changes in bladder cancer that related to disease prognosis [191], and Prdx3 upregulation was associated with increased cervical cancer risk [208]; H_2_O_2_-induced Prdx3 overexpression was observed in the eye lens in cataractogenesis [288]. Prdx4 was downregulated in acute promyelocytic leukemia [237] and Prdx3 overoxidization was reported in ageing [105]; moreover, Prdx3 was found to be downregulated in cells from Fanconi anaemia (FA-G) [57]. Unlike SOD-2, mitochondrial peroxiredoxins show different patterns of up- or downregulation in different disorders; however their pathogenetic roles deserve being elucidated in forthcoming studies of other OS/MDF-related disorders.

As another possibly sensitive endpoint in evaluating MDF, CoQ10 levels have been found lower than normal in an extensive number of disorders, including genetic diseases (Down syndrome, xeroderma pigmentosum, and Friedreich ataxia) [48, 73, 86], lung cancer [223], neurologic diseases (myalgic encephalomyelitis/chronic fatigue syndrome and Parkinson's sisease) [153, 162], and fibromyalgia [291]. These data, though confined to some selected disorders, concur with the most frequent findings of deficiencies in OXPHOS-related activities in most of the diseases where MDF was reported. It should be considered that measurements of CoQ10 levels in patients with OS/MDF-related disorders should be viewed as a preliminary step in view of possible clinical interventions using CoQ10.

Unlike OXPHOS, only few studies have reported on abnormalities in Krebs cycle-related endpoints. In Alzheimer's disease, a decreased expression of lipoamide dehydrogenase was detected in brain of both AD patients, and of AD mice, along with the observation of 4-HNE-induced oxidation of *α*-lipoic acid (ALA) [138]. Ageing was also associated with deficiencies in Krebs cycle-related endpoints, as tissue cells and skin fibroblasts of old donors displayed decreased expression of pyruvate dehydrogenase, pyruvate kinase, and lactate dehydrogenase [106]. A lipoic acid synthetase deficiency was detected in neonatal-onset epilepsy [149]. Quite surprisingly, no study was found in this review reporting on ALA levels, although analytical means for ALA measurement [308] and assay kits are available. This information gap should be filled in forthcoming studies both aimed at elucidating disease pathogenesis and in view of appropriate design for clinical trials using ALA.

Mitochondrial DNA was found to be affected by point mutations or by deletions both in primary and secondary mtDNA-related diseases (Table 1). Several other disorders, however, displayed mtDNA damage or changes, as mtDNA copy number or mitophagy [307]. A recent paper by Napoli el al. [171] reported on OS-mediated deletions mtDNA in autistic children; damaged and mutated mtDNA was detected in ageing cells [104–106]; among malignancies, breast, endometrial, gastric, hepatocellular, lung, and thyroid cancers were associated with dysregulation of mtDNA genes or of markers of mitochondrial biogenesis [199, 213, 214, 219, 228, 247, 248]; moreover, mtDNA damage was reported in cardiovascular disorders [112] and in osteoarthritis [122–124]. Other two markers of MDF, either mitochondrial membrane potential (ΔΨ) or mitochondrial ultrastructure, were found to be altered in several disorders, including Fanconi anaemia [57–59], Down syndrome (see [49, 52], reviewed in [309]), and fibromyalgia [291, 292].

Far from being confined to the classically termed mitochondrial diseases, the present information points to the association of MDF with OS and, to a limited extent, with NS across a number of diseases pertaining to broad-ranging medical disciplines. Thus, one may imagine treasuring the current knowledge of mitochondrial diseases in order to design appropriate interventions in several pathologies. In this prospect, the cofactors recognized as “mitochondrial nutrients,” as ALA, CoQ10, and l-carnitine (CARN) (or its derivatives) [310, 311] should be regarded as a prime tool in mitigating MDF and, hence, OS in several disorders; a line of ongoing clinical studies is foreseen by at least one extensive study group focused on mitochondrial diseases, on the grounds of the novel discipline termed “mitochondriology” [312, 313]. The present and growing awareness for the pathogenetic roles of OS/MDF in several disorders has prompted a number of clinical groups to undertake clinical trials testing mitochondrial nutrients aimed at counteracting OS/MDF in patients with an extensive number of diseases. The current knowledge of previous clinical trials and the prospects of study design are reported in a parallel review to the present paper [314].

## 10. Conclusion

The association of MDF with OS and, probably, NS is a widespread phenomenon encompassing a broad number of different disorders. The current knowledge of mitochondrial diseases and of other OS/MDF related diseases should prompt further investigations to elucidate the roles of OS/MDF in a number of disorders, in the prospect of rational interventions aimed at mitigating OS/MDF-related clinical progression.

## Figures and Tables

**Table 1:** Mitochondrial dysfunction (MDF) and/or oxidative stress (OS) in some selected mitochondrial diseases.

	MDF/OS endpoints	References
Primary mitochondrial DNA-related diseases		
Leber's hereditary optic neuropathy (LHON)	mtDNA point mutations; Idebenone- and EPI-743-induced protection	[9–11]
Leigh syndrome, subacute necrotizing encephalomyelopathy	mtDNA point mutations; EPI-743-induced protection	[12]
Neuropathy, ataxia, retinitis pigmentosa, and ptosis (NARP)	↓ ATP synthase activity, ↑ ROS; ↑ p66Shc phosphorylation; antioxidants-induced protection	[13, 14]
Mitochondrial myopathy, encephalomyopathy, lactic acidosis, stroke-like symptoms (MELAS)	mtDNA Point Mutations; ↓ Complex I, III And IV, ↑ Free radicals in CSF, CoQ10-induced protection	[15–19]
Myoclonic epilepsy with ragged red fibers (MERRF)	mtDNA point mutations; ↓ ATP, ↑ Matrix metallo-proteinase 1; ↑ ROS, ↑ carbonylated mt proteins	[20, 21]
Maternally inherited diabetes mellitus and deafness (MIDD)	mtDNA point mutations; CoQ10-induced protection	[22]
Kearns-Sayre syndrome (KSS)	mtDNA deletions; ↑ Myoglobin and antioxidant enzymes mtDNA deletions with cyt-c oxidase-deficient cells and loss of mitochondrial respiratory chain subunits	[23]
Chronic progressive external ophthalmoplegia (CPEO)	mtDNA deletions and point mutations; ↓ Complex I and IV; ↑ OS biomarkers; tetracycline-induced antioxidant protection	[24, 25]
Pearson syndrome	mtDNA deletions; ↓ OXPHOS, iron overload	[26, 27]

Secondary mitochondrial DNA-related diseases		
Alpers-Huttenlocher Syndrome	Pol *γ* mutations; ↓ OXPHOS; mtDNA deletions/depletion	[28–31]
Mitochondrial neurogastrointestinal encephalomyopathy (MNGIE)	Thymidine phosphorylase mutation; mtDNA deletions ↓ CoQ10 levels	[32, 33]

**Table 2:** Genetic diseases displaying OS/MDF hallmarks.

Genetic diseases	OS/MDF-related Endpoints	References
Cancer-prone and/or early ageing diseases		
Ataxia-telangiectasia	↑ ROS production; abnormal mt structure; ↓ΔΨ; ↑ mt enzymes; ↓ mitophagy	[35–39]
Bloom syndrome	↑ ROS production; antioxidant sensitive; ↑ WBC 8-OHdG	[40–42]
Cockayne syndrome	↓ OGG1; ↑ ROS production; ↑ DNA oxidative damage; ↑ mitophagy	[43–46]
	defective CSA and CSB localize to mt →↓ interaction with mt OGG1	
Down syndrome	↑ ROS production; affected mt structure; defective Complex I activity;	[47–52]
	↓ CoQ10 lymphocyte and platelet levels; sensitive to CoQ10	
Fanconi anaemia	↑ ROS production; ↑ 8-OHdG; ↓ GSH:GSSG; ↑ methylglyoxal; antioxidant	[54–60]
	sensitive; redox functions of FANC proteins; downregulation of antioxidant,	
	chelating and stress proteins; ↓ ATP; ↓ΔΨ; ↓ Prdx3; abnormal mt structure	
Hutchinson-Gilford syndrome	↑ ROS production; ↑ SOD-2 transcript; ↓ ATP content; ↓ caspase-like	[61, 62]
	proteasome activity; NAC sensitive	
Nijmegen breakage syndrome	PARP hyperactivation and ↑ ROS production; ↓ mt p53 translocation	[63, 64]
Rothmund-Thomson syndrome	RECQL4 response to OS; RECQL4 interaction with PARP-1 and p53	[65–67]
Werner Syndrome	WRN regulates HIF-1 activation inducing mt ROS; ↑ OS; abnormal mt structure	[68–72]
Xeroderma pigmentosum	↓ repair of cyclo-dA; ↑ lipid peroxidation and protein glycation; ↓ CoQ serum levels;	[73–77]
	defective mt gene transcripts for 16 S rRNA, ATPase 6L and lactate dehydrogenase	

Neurological and muscle genetic diseases		
Adrenoleukodystrophy	↑ mtDNA oxidation and impaired OXPHOS; ↓ complex V; ↓ GSH; ↑ GSSG	[78–82]
	↓ total antioxidant defenses in symptomatic but not in asymptomatic patients;	
	mitochondrial inner membrane potential dissipation; ↓ ATP	
	ATP/ADP ratio; abnormal mt ultrastructure; dysregulated Fe metabolism	
Duchenne Muscular Dystrophy	↑ protein thiol oxidation; ↑ lipofuscin; ↑ 4-hydroxynonenal; ↑ total hydroperoxides;	[83–85]
	uncoupled OXPHOS; ↓ maximal ATP synthesis; ↓ *γ*-glutamyl cysteine ligase and GSH	
Friedreich Ataxia	↓ complex I, II and III; ↓ aconitase; ↓ CoQ10 and Vit E; ↓ Fe-S cluster	[86–88]
	biosynthesis; mt Fe overload and cellular Fe dysregulation; ↑ sensitivity to OS	
Huntington's Disease	CoQ10-induced ↓ brain protein carbonyls; ↑ NADPH oxidase (NOX) activity in	[89–92]
	human HD brains; CoQ10 + creatine exert additive neuroprotective effects in HD mice and rats	

Other Genetic Diseases		
Hyperhomocysteinaemia	↑ MDA levels and carbonyl formation; ↓ sulfhydryl groups and total antioxidant	[93–95]
	status; ↓ΔΨ; release of cytochrome-c; ↑ mt matrix metalloproteinase	
Sickle Cell Disease	↑ ROS production; ↑ advanced glycation end products (AGEs); ↓ GSH Iron-laden mt in WBC	[96–98]
Thalassaemia	Non-transferrin-bound iron (NTBI) → damage to mitochondria, lysosomes,	[99–101]
	lipid membranes, proteins, and DNA; ↑ WBC 8-OHdG; ↓ΔΨ; ↓ carnitine	

**Table 3:** Ageing and ageing-related degenerative disorders displaying OS/MDF hallmarks.

Diseases	OS/MDF hallmarks	References
Ageing	Age-related ↑ oxidative DNA damage and ↓ GSH : GSSG ratio; ↑ Fe accumulation;	[102–107]
	impaired mt function related to ↓ complex I and IV; overoxidized Prdx3; mtDNA damage	
	↓ pyruvate dehydrogenase; ↑ pyruvate kinase and lactate dehydrogenase	

Cardiovascular diseases	lipid and protein oxidation; NADPH oxidase dysregulation; ↑ peroxynitrite;	[108–113]
	mt damage and dysfunction; endothelial dysfunction, with ↓ NO bioactivity;	
	↑ SOD-2 and mtDNA mutations in atherosclerotic plaques	

Metabolic syndrome	↑ plasma 8-isoprostanes; ↓ total antioxidant capacity; ↓ serum Vit C and E;	[114–119]
	↑ TBARS; ↓ complex I; ↑ NADPH oxidase; downregulated SIRT3 leading to	
	↑ mt protein acetylation; Nod-like receptor protein 3 (NLRP3) activation; ↓ mtDNA	
	copy number; ↓ paraoxonase-1; dysregulated carnitine palmitoyltransferase	

Osteoarthritis	↓ total antioxidant capacity, thiol levels, catalase activity and prolidase activities;	[120–124]
	↑ total peroxides and lipid peroxides, ↑ myeloperoxidase; mt genome dysregulation	
	(17 up- and 9 downregulated mt genes); ↓ complexes I, II and III; ↑↓ SOD-2	

Type 2 diabetes mellitus	↑ 15-F2t-IsoP and AOPP; ↑ HNE, Trx and HSP70; ↓ GSH:GSSG; ↑ 8-OHdG and	[125–133]
	protein carbonyls in saliva; ↑ NADPH oxidase; ↓ complex I and/or IV;	
	↑ SOD-2 and TrxR3 in platelets; ↑ NO and S-nitrosylation	

**Table 4:** Neurologic and neuropsychiatric diseases displaying OS/MDF hallmarks.

Diseases	OS/MDF hallmarks	References
A. neurologic diseases		
Alzheimer's Disease	Aberrations in OXPHOS and Krebs cycle; altered mt-associated endoplasmic	[134–141]
	reticulum membranes as related to amyloid precursor protein; dysregulated Fe	
	metabolism; HNE- induced oxidation of lipoic acid; ↓ lipoamide dehydrogenase in	
	brain of AD patients and of AD mice; ↓ Complexes III and IV; ↑ mitochondrial mass	
Amyotrophic lateral sclerosis	protein misfolding at endoplasmic reticulum (ER); altered Golgi network; oxidative	[142–146]
	damage to ER proteins; ↓ WBC glutathione peroxidase; ↓ SOD-1 related to disease	
	progression; ↓ NADPH oxidase; ↓ complex I and ATP/ADP ratio; abnormal mt ultrastructure	
Epilepsy	↑ NOS II with NO-, ^∙^O_2_^−^ and ONOO^−^ dependent ↓ complex I activity; ↑ complex	[147–150]
	III-dependent ^∙^O_2_^−^ production of brain mitochondria; ↓ lipoic acid synthetase	
Myalgic encephalomyelitis/ /chronic fatigue syndrome	↑ urinary 8-OHdG; ↑ TBARS; ↓ Vit C; ↓ HSP70; ↑ plasma peroxides and oxidized	[151–156]
	LDL; ↓ CoQ10; ↑ ATP production; ↑ complex I, III and IV	
Multiple sclerosis	↑ lipid hydroperoxide, carbonyl protein, and NO metabolites; ↓ total radical-trapping	[157–160]
	antioxidant parameter; Melatonin-induced ↑ SOD and ↑ GPx, and ↓ MDA; ↓ OXPHOS	
	activity; mtDNA deletions	
Parkinson's disease	↑ lipid peroxides; ↑ urinary 8-OHdG; ↑ plasma F(2)-IsoPs, HETEs, 7beta-and	[6, 161–165]
	27-hydroxycholesterol, 7-ketocholesterol, F(4)-NPs; dysregulated Fe metabolism	
	↓ CoQ10, and ↓ complex V; ↑↓ cysteine oxidation within complex I; ↑ lactate	

B. psychiatric diseases		
Autistic spectrum disorders	↓ GSH/GSSG in WBC; ↑ 4-hydroxynonenal in plasma and RBC membranes;	[166–172]
	↑ 3-nitrotyrosine and 8-OHdG; downregulated OXPHOS (↓ complex I, III, IV and	
	V); ↓ aconitase; ↑ mtDNA deletions, GC→AT transitions, and GC→TA transitions	
Bipolar disorder	↓ attachment of hexokinase 1 to outer mt membrane;	[173–175]
	↓ GSH/GSSG; ↓ CAT expression;	
Major depression	altered IL-6, IL-10, and IL-6/IL-10 ratio versus F2-isoprostanes; ↑ protein	[176–179]
	carbonylation and glutathione reductase expression; ↑ SOD, CAT and PER	
	↑ PMNs apoptosis (cytochrome c release); ↓ complex I subunits	
Obsessive-compulsive disorder	↑ MDA/TBARS; ↑ SOD; ↓ GSH/GSSG; prevailing CC (Ala/Ala) SOD-2 genotype	[180–182]
Schizophrenia	↑ lipid peroxidation, damage to proteins, and DNA; ↓ antioxidant levels (CoQ10,	[183–187]
	Vit E, GSH and melatonin); autoimmune responses; ↑ TBARS, IL-6 and PCC levels;	
	↓ TRX levels; ↓ plasma total antioxidant status	

**Table 5:** Neoplastic disorders displaying OS/MDF hallmarks.

Malignancies	OS/MDF hallmarks	References
Bladder cancer	↑ 8-OHdG, 3-NT, and Prdx4 associated with a poor prognosis; ↓ serum levels of	[191–194]
	Vit C and E, SOD and GPx, and serum antioxidant capacity; ↑ mtDNA mutations;	
	altered mitochondrial protein expression profile (OXPHOS, glycolysis/	
	/gluconeogenesis, Krebs cycle)	

Breast cancer	↑ 8-OH-dG and protein carbonyl; ↑ total SOD, SOD-1, and EC-SOD in plasma	[195–201]
	and breast tumours; BRCA1 mutations cause OS in tumour microenvironment;	
	↑ >95 gene transcripts associated with mt biogenesis and/or mt translation;	
	mt location of phosphorylated BRCA1; progression-related ↓ CoQ10 levels; ↓ SOD-2	

Cervical cancer	↑ lipid peroxidation; ↑ GPx; ↓ SOD; ↓ CAT; null GSTM1 and GSTT1	[202–208]
	polymorphisms associated with ↑ risk of cervical neoplasia; ↑ Prdx3	

Colorectal cancer	↑ 8-OHdG; ↑ SOD, GR and GPx; ↓ GST; U-shaped association between	[209–211]
	the relative mtDNA copy number and risk of colorectal cancer	

Endometrial cancer	↑ tumour specific mtDNA mutations; ↓ complex I; ↑ mtDNA level;↑ mitochondrial	[212–214]
	biogenesis; ↑ SOD-2; ↑ CAT; ↑ Prx3	

Gastric cancer	↑ 8-OHdG; ↓ hOGG1; ↑ SOD-2; *H. pylori*-induced mtDNA mutations and decrease	[215–217]
	of mtDNA content	

Hepatocellular carcinoma growth	↑ 8-OHdG and 4-HNE associated with microvessel density, vascular endothelial	[218–221]
	Factor, and Akt activity; d-ROM, *α*-fetoprotein, and fasting plasma associated with HCC	
	recurrence; survival rate related to ↑ SOD-2 and Trx; ↑ mtDNA 9-bp polymorphism	

Lung cancer	↑ MDA; ↑ aldehydes in exhaled breath; ↑ 8-OHdG; polymorphism of OGG1	[222–228]
	Ser326Cys; ↑ thymidine glycol in ever-smokers; ↓ CAT and carbonic anhydrase;	
	↓ AOC in never-smokers than in ever-smokers; ↑ HO-1 expression;	
	↓ CoQ10; mt HSP90s associates with worse clinical outcome; ↓ mtDNA; ↑ ΔΨm	

Melanoma	↑ 8OHdG associated with a poor prognosis; ↑ NOS1 polymorphism (rs2682826);	[229–232]
	↑ TNF-*α* enhancing action of tumour-associated macrophages; ↑ OXPHOS	

Myeloid leukaemias	↑ MDA; ↓ thiol plasma levels; ↑ PC, TBARS and LOOH; ↓ ferric reducing ability	[233–241]
	of plasma (FRAP); GSTP1 Ile105Val polymorphism associated with CML; ↑ CAT;	
	↑ Trx; ↑ adenosine deaminase and xanthine oxidase; ↓ PRDX4 expression in APL;	
	disease-related 2- to 50-fold ↑ amplification of mtDNA; survival advantage in patients	
	with SOD-2 T versus C polymorphism; ↓ mt-encoded genes and ↑ mtDNA copy number	

Oral cancer	↑ MDA and NO; ↑ AGEs; ↓ CAT ↓ SOD in tissue, while ↑ SOD in erythrocytes;	[242–246]
	somatic mutation of D-loop of mtDNA was associated with better survival	

Thyroid oncocytic carcinoma	disruptive mtDNA mutations in complex I and III; ↓ complex I; ↓ respiration;	[247, 248]
	↓ ATP synthesis; ↑ ROS; ↓ mitochondrial biogenesis	

**Table 6:** Autoimmune diseases displaying OS/MDF hallmarks.

Diseases	OS/MDF hallmarks	References
Antiphospholipid Syndrome	↑ Serum amyloid A, C-reactive protein, and 8-isoprostane and prostaglandin E2	[249, 250]
	↑ peroxide production, nuclear abundance of Nrf2, ↓ antioxidant enzymatic activity;	
	↓ intracellular GSH; altered ΔΨ; beneficial effects of CoQ10 supplementation	

Pemphigus Vulgaris	↑ MDA; ↓ antioxidants (Vit. A and E); ↓↑ CAT and ↓ GSH-Px; autoantibodies	[251–254]
	against desmosomal, mitochondrial, and other keratinocyte self-antigens	

Primary Biliary Cirrhosis	↓ Antioxidant vitamins (A, C and E); ↓ GST, ↑ HOCl associated with eosinophil	[255–258]
	peroxidase; ↑ IgG against LOOH; serum and saliva antimitochondrial autoantibodies	
	against E2 subunits of 2-oxoacid dehydrogenase enzymes (pyruvate dehydrogenase,	
	2-oxo-acid dehydrogenase, and 2-oxo-glutarate dehydrogenase)	

Psoriasis	↑ MDA, 8-OHdG, LOOH and NOx; ↓ CAT, SOD, GSH-Px, GSH and total	[259–263]
	antioxidant status; release of mitochondrial cytochrome c; ↑ CYP4F8 mRNA	

Rheumatoid Arthritis	↑ LOOH; ↑ MPO, ↑ ^•^O_2_^−^ and ^•^OH in neutrophils from peripheral blood and	[264–269]
	synovial infiltrate; ↓ CAT, SOD, GSH-Px, GSH; TNF-*α*-associated ER stress;	
	↑ AGE-IgG; chemokine (MCP-1/CCL2, RANTES/CCL5)-associated ROS;	
	mtDNA mutations positively correlated with macroscopic synovitis	

Sjøgren's Syndrome	↑ protein carbonyl and AGEs; ↑ 4-HNE; ↑ TNF-*α*, MPO and nitrotyrosine;	[270–274]
	↑ 8-OHdG and hexanoyl-lysine; ↑ Trx; ↑ LDH and mitochondrial GOT;	
	antimitochondrial autoantibodies in patients' saliva	

Systemic Lupus Erythematosus	↓ CAT, SOD, GSH-Px, GSH; ↑ chemokine-associated ROS;	[264, 275, 276]
	↓ ATP; ↑ ΔΨ; persistent mt hyperpolarization	
	abnormal expression of mt pyruvate dehydrogenase complex	

Systemic Sclerosis	↑ MDA, dityrosines and carbonyls; ↓ uric acid and total antioxidants status in	[277–280]
	saliva; ↓ GSH; ↓ SOD; ↑ GPx; plasma antimitochondrial autoantibodies	

Vitiligo	↑ 4-HNE; ↓ cardiolipin; ↓ GSH; ↓ MPO; ↓ GST and CAT; ↑ GSTM1 null and	[281–283]
	GSTT1/GSTM1 double-null type; altered OXPHOS lipid-dependent subunits	

**Table 7:** Miscellaneous pathologies displaying OS/MDF hallmarks.

Diseases/conditions	OS/MDF hallmarks	References
Cataract	↑ TBARS; ↓ thiols; ↓ ferric reducing/antioxidant power;	[284–288]
	↑ dehydroascorbate/ascorbate; ↑ G/G genotype of SOD-1—251A/G	
	polymorphism; H_2_O_2_-specific induction of Prdx3 in the eye lens	

Fibromyalgia	↑ lipid peroxidation; ↓ total antioxidant capacity; ↑ TNF-*α*;	[289–293]
	↓ ΔΨ; ↓ CoQ10; ↑ mt ^•^O_2_^−^; excess mitophagy	

Malformations	OS and MDF in teratogenic action; thalidomide causes embryonic DNA	[294–300]
	oxidation in susceptible but not resistant species; GSH depletion, or inhibition	
	of GPx or GR; ↓ mt NADH oxidase in teratogen-sensitive embryonic tissues;	
	ethanol upregulates NOX regulatory subunits and increases NOX expression;	
	↓ complex I and IV and ATP synthase in fetal alcohol syndrome	

## References

[1] Vitols E, Linnane AW (1961). Studies on the oxidative metabolism of *Saccharomyces cerevisiae*. II. Morphology and oxidative phosphorylation capacity of mitochondria and derived particles from baker's yeast. *The Journal of Biophysical and Biochemical Cytology*.

[2] Bellamy D (1962). The endogenous citric acid-cycle intermediates and amino acids of. *The Biochemical Journal*.

[3] Richter C, Kass GEN (1991). Oxidative stress in mitochondria: its relationship to cellular Ca^2+^ homeostasis, cell death, proliferation and differentiation. *Chemico-Biological Interactions*.

[4] Sohal RS, Brunk UT (1992). Mitochondrial production of pro-oxidants and cellular senescence. *Mutation Research—DNAging Genetic Instability and Aging*.

[5] Piccolo G, Banfi P, Azan G (1991). Biological markers of oxidative stress in mitochondrial myopathies with progressive external ophthalmoplegia. *The Journal of the Neurological Sciences*.

[6] Di Monte DA, Chan P, Sandy MS (1992). Glutathione in Parkinson’s disease: a link between oxidative stress and mitochondrial damage?. *Annals of Neurology*.

[7] Chinnery PF, Pagon RA, Bird TD, Dolan CR (2010). Mitochondrial disorders overview. Synonyms: mitochondrial encephalomyopathies, mitochondrial myopathies, oxidative phosphorylation disorders, respiratory chain disorders. *GeneReviews*.

[8] Schon EA, di Mauro S, Hirano M (2012). Human mitochondrial DNA: roles of inherited and somatic mutations. *Nature Reviews Genetics*.

[9] Carelli V, La Morgia C, Valentino ML (2011). Idebenone treatment in Leber’s hereditary optic neuropathy. *Brain*.

[10] Kumar M, Kaur P, Kumar M (2012). Clinical characterization and mitochondrial DNA sequence variations in Leber hereditary optic neuropathy. *Molecular Vision*.

[11] Sadun AA, Chicani CF, Ross-Cisneros FN (2012). Effect of EPI-743 on the clinical course of the mitochondrial disease leber hereditary optic neuropathy. *Archives of Neurology*.

[12] Martinelli D, Catteruccia M, Piemonte F (2012). EPI-743 reverses the progression of the pediatric mitochondrial disease—genetically defined Leigh Syndrome. *Molecular Genetics and Metabolism*.

[13] Lebiedzinska M, Karkucinska-Wieckowska A, Wojtala A (2013). Disrupted ATP synthase activity and mitochondrial hyperpolarisation-dependent oxidative stress is associated with p66Shc phosphorylation in fibroblasts of NARP patients. *International Journal of Biochemistry and Cell Biology*.

[14] Mattiazzi M, Vijayvergiya C, Gajewski CD (2004). The mtDNA T8993G (NARP) mutation results in an impairment of oxidative phosphorylation that can be improved by antioxidants. *Human Molecular Genetics*.

[15] Ihara Y, Kibata M, Hayabara T (1997). Free radicals in the cerebrospinal fluid are associated with neurological disorders including mitochondrial encephalomyopathy. *Biochemistry and Molecular Biology International*.

[16] Vattemi G, Mechref Y, Marini M (2011). Increased protein nitration in mitochondrial diseases: evidence for vessel wall involvement. *Molecular and Cellular Proteomics*.

[17] Tan TMM, Caputo C, Medici F (2009). MELAS syndrome, diabetes and thyroid disease: the role of mitochondrial oxidative stress. *Clinical Endocrinology*.

[18] Cotán D, Cordero MD, Garrido-Maraver J (2011). Secondary coenzyme Q(10) deficiency triggers mitochondria degradation by mitophagy in MELAS fibroblasts. *The FASEB Journal*.

[19] Katayama Y, Maeda K, Iizuka T (2009). Accumulation of oxidative stress around the stroke-like lesions of MELAS patients. *Mitochondrion*.

[20] Ma Y, Chen Y, Lu C, Liu C, Wei Y (2005). Upregulation of matrix metalloproteinase 1 and disruption of mitochondrial network in skin fibroblasts of patients with MERRF syndrome. *Annals of the New York Academy of Sciences*.

[21] Wu S, Ma Y, Wu Y, Chen Y, Wei Y (2010). Mitochondrial DNA mutation-elicited oxidative stress, oxidative damage, and altered gene expression in cultured cells of patients with MERRF syndrome. *Molecular Neurobiology*.

[22] Salles JE, Moisés VA, Almeida DR, Chacra AR, Moisés RS (2006). Myocardial dysfunction in mitochondrial diabetes treated with Coenzyme Q10. *Diabetes Research and Clinical Practice*.

[23] Lax NZ, Campbell GR, Reeve AK (2012). Loss of myelin-associated glycoprotein in kearns-sayre syndrome. *Archives of Neurology*.

[24] Mancuso M, Orsucci D, Calsolaro V (2011). Tetracycline treatment in patients with progressive external ophthalmoplegia. *Acta Neurologica Scandinavica*.

[25] Wolf J, Obermaier-Kusser B, Jacobs M (2012). A new mitochondrial point mutation in the transfer RNALys gene associated with progressive external ophthalmoplegia with impaired respiratory regulation. *The Journal of the Neurological Sciences*.

[26] Manea EM, Leverger G, Bellmann F (2009). Pearson syndrome in the neonatal period: two case reports and review of the literature. *Journal of Pediatric Hematology/Oncology*.

[27] Kefala-Agoropoulou K, Roilides E, Lazaridou A (2007). Pearson syndrome in an infant heterozygous for C282Y allele of HFE gene. *Hematology*.

[28] Uusimaa J, Finnilä S, Vainionpää L (2003). A mutation in mitochondrial DNA-encoded cytochrome c oxidase II gene in a child with Alpers-Huttenlocher-like disease. *Pediatrics*.

[29] de Vries MC, Rodenburg RJ, Morava E (2007). Multiple oxidative phosphorylation deficiencies in severe childhood multi-system disorders due to polymerase gamma (POLG1) mutations. *European Journal of Pediatrics*.

[30] Lewis W, Day BJ, Kohler JJ (2007). Decreased mtDNA, oxidative stress, cardiomyopathy, and death from transgenic cardiac targeted human mutant polymerase *γ*. *Laboratory Investigation*.

[31] Saneto RP, Cohen BH, Copeland WC (2013). Alpers-Huttenlocher Syndrome. *Pediatric Neurology*.

[32] Hirano M, Garone C, Quinzii CM (2012). CoQ10 deficiencies and MNGIE: two treatable mitochondrial disorders. *Biochimica et Biophysica Acta—General Subjects*.

[33] El-Hattab AW, Scaglia F (2013). Mitochondrial DNA depletion syndromes: review and updates of genetic basis, manifestations, and therapeutic options. *Neurotherapeutics*.

[34] DiMauro S, Schon EA, Carelli V (2013). The clinical maze of mitochondrial neurology. *Nature Reviews Neurology*.

[35] Barzilai A, Rotman G, Shiloh Y (2002). ATM deficiency and oxidative stress: a new dimension of defective response to DNA damage. *DNA Repair*.

[36] Ambrose M, Goldstine JV, Gatti RA (2007). Intrinsic mitochondrial dysfunction in ATM-deficient lymphoblastoid cells. *Human Molecular Genetics*.

[37] Degan P, d’Ischia M, Pallardó FV (2007). Glutathione levels in blood from ataxia telangiectasia patients suggest in vivo adaptive mechanisms to oxidative stress. *Clinical Biochemistry*.

[38] Valentin-Vega YA, MacLean KH, Tait-Mulder J (2012). Mitochondrial dysfunction in ataxia-telangiectasia. *Blood*.

[39] Shiloh Y, Tabor E, Becker Y (1983). Abnormal response of ataxia-telangiectasia cells to agents that break the deoxyribose moiety of DNA via a targeted free radical mechanism. *Carcinogenesis*.

[40] Poot M, Hoehn H, Nicotera TM, Rudiger HW (1989). Cell kinetic evidence suggests elevated oxidative stress in cultured cells of Bloom’s syndrome. *Free Radical Research Communications*.

[41] Nicotera TM (1991). Molecular and biochemical aspects of Bloom’s syndrome. *Cancer Genetics and Cytogenetics*.

[42] Zatterale A, Kelly FJ, Degan P (2007). Oxidative stress biomarkers in four Bloom syndrome (BS) patients and in their parents suggest in vivo redox abnormalities in BS phenotype. *Clinical Biochemistry*.

[43] Javeri A, Guy Lyons J, Huang XX, Halliday GM (2011). Downregulation of Cockayne syndrome B protein reduces human 8-oxoguanine DNA glycosylase-1 expression and repair of UV radiation-induced 8-oxo-7,8-dihydro-2′-deoxyguanine. *Cancer Science*.

[44] Pascucci B, Lemma T, Iorio E (2012). An altered redox balance mediates the hypersensitivity of Cockayne syndrome primary fibroblasts to oxidative stress. *Aging Cell*.

[45] Scheibye-Knudsen M, Ramamoorthy M, Sykora P (2012). Cockayne syndrome group B protein prevents the accumulation of damaged mitochondria by promoting mitochondrial autophagy. *The Journal of Experimental Medicine*.

[46] Kamenisch Y, Berneburg M (2013). Mitochondrial CSA and CSB: protein interactions and protection from ageing associated DNA mutations. *Mechanisms of Ageing and Development*.

[47] Pallardó FV, Degan P, d’Ischia M (2006). Higher age-related prooxidant state in young Down syndrome patients indicates accelerated aging. *Biogerontology*.

[48] Tiano L, Padella L, Carnevali P (2008). Coenzyme Q(10) and oxidative imbalance in Down syndrome: biochemical and clinical aspects. *BioFactors*.

[49] Tiano L, Busciglio J (2011). Mitochondrial dysfunction and Down’s syndrome: is there a role for coenzyme Q(10)?. *BioFactors*.

[50] Valenti D, Manente GA, Moro L, Marra E, Vacca RA (2011). Deficit of complex I activity in human skin fibroblasts with chromosome 21 trisomy and overproduction of reactive oxygen species by mitochondria: involvement of the cAMP/PKA signalling pathway. *Biochemical Journal*.

[51] Valenti D, de Rasmo D, Signorile A (2013). Epigallocatechin-3-gallate prevents oxidative phosphorylation deficit and promotes mitochondrial biogenesis in human cells from subjects with Down's syndrome. *Biochimica et Biophysica Acta*.

[52] Bambrick LL, Fiskum G (2008). Mitochondrial dysfunction in mouse trisomy 16 brain. *Brain Research*.

[53] Brooksbank BW, Balazs R (1984). Superoxide dismutase, glutathione peroxidase and lipoperoxidation in Down’s syndrome fetal brain. *Brain Research*.

[54] Joenje H, Arwert F, Eriksson AW (1981). Oxygen-dependence of chromosomal aberrations in Fanconi’s anaemia. *Nature*.

[55] Pagano G, Aiello Talamanca A, Castello G (2013). From clinical description to in vitro and animal studies, and backwards to patients: oxidative stress and mitochondrial dysfunction in Fanconi anemia. *Free Radical Biology and Medicine*.

[56] Du W, Rani R, Sipple J (2012). The FA pathway counteracts oxidative stress through selective protection of antioxidant defense gene promoters. *Blood*.

[57] Mukhopadhyay SS, Leung KS, Hicks MJ, Hastings PJ, Youssoufian H, Plon SE (2006). Defective mitochondrial peroxiredoxin-3 results in sensitivity to oxidative stress in Fanconi anemia. *The Journal of Cell Biology*.

[58] Kumari U, Jun WY, Bay BH (2013). Evidence of mitochondrial dysfunction and impaired ROS detoxifying machinery in Fanconi anemia cells. *Oncogene*.

[59] Ravera S, Vaccaro D, Cuccarolo P (2013). Mitochondrial respiratory chain Complex I defects in Fanconi anemia complementation group A. *Biochimie*.

[60] Pagano G, Aiello Talamanca A, Castello G (2013). Bone marrow cell transcripts from Fanconi anaemia patients reveal in vivo alterations in mitochondrial, redox and DNA repair pathways. *European Journal of Haematology*.

[61] Viteri G, Chung YW, Stadtman ER (2010). Effect of progerin on the accumulation of oxidized proteins in fibroblasts from Hutchinson Gilford progeria patients. *Mechanisms of Ageing and Development*.

[62] Richards SA, Muter J, Ritchie P, Lattanzi G, Hutchison CJ (2011). The accumulation of un-repairable DNA damage in laminopathy progeria fibroblasts is caused by ROS generation and is prevented by treatment with N-acetyl cysteine. *Human Molecular Genetics*.

[63] Turinetto V, Porcedda P, Minieri V (2010). A novel defect in mitochondrial p53 accumulation following DNA damage confers apoptosis resistance in Ataxia Telangiectasia and Nijmegen Breakage Syndrome T-cells. *DNA Repair*.

[64] Krenzlin H, Demuth I, Salewsky B (2012). DNA damage in nijmegen breakage syndrome cells leads to PARP hyperactivation and increased oxidative stress. *PLoS Genetics*.

[65] Woo LL, Futami K, Shimamoto A, Furuichi Y, Frank KM (2006). The Rothmund-Thomson gene product RECQL4 localizes to the nucleolus in response to oxidative stress. *Experimental Cell Research*.

[66] Davis T, Tivey HS, Brook AJ (2013). Activation of p38 MAP kinase and stress signalling in fibroblasts from the progeroid Rothmund-Thomson syndrome. *Age*.

[67] Croteau DL, Rossi ML, Canugovi C (2012). RECQL4 localizes to mitochondria and preserves mitochondrial DNA integrity. *Aging Cell*.

[68] Pallardó FV, Lloret A, Lebel M (2010). Mitochondrial dysfunction in some oxidative stress-related genetic diseases: Ataxia-Telangiectasia, Down Syndrome, Fanconi Anaemia and Werner Syndrome. *Biogerontology*.

[69] Bohr VA, Cooper M, Orren D (2000). Werner syndrome protein: biochemical properties and functional interactions. *Experimental Gerontology*.

[70] von Kobbe C, May A, Grandori C, Bohr VA (2004). Werner syndrome cells escape hydrogen peroxide-induced cell proliferation arrest. *The FASEB Journal*.

[71] Pagano G, Zatterale A, Degan P (2005). Multiple involvement of oxidative stress in Werner syndrome phenotype. *Biogerontology*.

[72] Lloret A, Calzone R, Dunster C (2008). Different patterns of in vivo pro-oxidant states in a set of cancer- or aging-related genetic diseases. *Free Radical Biology and Medicine*.

[73] Hayashi M (2008). Roles of oxidative stress in xeroderma pigmentosum. *Advances in Experimental Medicine and Biology*.

[74] Ting APL, Low GKM, Gopalakrishnan K, Hande MP (2010). Telomere attrition and genomic instability in xeroderma pigmentosum type-b deficient fibroblasts under oxidative stress. *Journal of Cellular and Molecular Medicine*.

[75] Rezvani HR, Rossignol R, Ali N (2011). XPC silencing in normal human keratinocytes triggers metabolic alterations through NOX-1 activation-mediated reactive oxygen species. *Biochimica et Biophysica Acta—Bioenergetics*.

[76] Tanaka J, Nagai T, Okada S (1998). Serum concentration of coenzyme Q in xeroderma pigmentosum. *Clinical Neurology*.

[77] Xia X, Werner D, Popanda O, Thielmann HW (1994). Expression of mitochondrial genes and DNA-repair-related nuclear genes is altered in xeroderma pigmentosum fibroblasts. *The Journal of Cancer Research and Clinical Oncology*.

[78] Deon M, Sitta A, Barschak AG (2007). Induction of lipid peroxidation and decrease of antioxidant defenses in symptomatic and asymptomatic patients with X-linked adrenoleukodystrophy. *International Journal of Developmental Neuroscience*.

[79] Petrillo S, Piemonte F, Pastore A (2013). Glutathione imbalance in patients with X-linked adrenoleukodystrophy. *Molecular Genetics and Metabolism*.

[80] Schlüter A, Espinosa L, Fourcade S (2012). Functional genomic analysis unravels a metabolic-inflammatory interplay in adrenoleukodystrophy. *Human Molecular Genetics*.

[81] López-Erauskin J, Galino J, Ruiz M (2013). Impaired mitochondrial oxidative phosphorylation in the peroxisomal disease X-linked adrenoleukodystrophy. *Human Molecular Genetics*.

[82] Fourcade S, López-Erauskin J, Ruiz M (2014). Mitochondrial dysfunction and oxidative damage cooperatively fuel axonal degeneration in X-linked adrenoleukodystrophy. *Biochimie*.

[83] Terrill JR, Radley-Crabb HG, Iwasaki T (2013). Oxidative stress and pathology in muscular dystrophies: focus on protein thiol oxidation and dysferlinopathies. *FEBS Journal*.

[84] Renjini R, Gayathri N, Nalini A, Srinivas Bharath MM (2012). Oxidative damage in muscular dystrophy correlates with the severity of the pathology: role of glutathione metabolism. *Neurochemical Research*.

[85] Percival JM, Siegel MP, Knowels G (2013). Defects in mitochondrial localization and ATP synthesis in the mdx mouse model of Duchenne muscular dystrophy are not alleviated by PDE5 inhibition. *Human Molecular Genetics*.

[86] Cooper JM, Korlipara LVP, Hart PE, Bradley JL, Schapira AHV (2008). Coenzyme Q(10) and vitamin E deficiency in Friedreich’s ataxia: predictor of efficacy of vitamin e and coenzyme Q(10) therapy. *The European Journal of Neurology*.

[87] Sparaco M, Gaeta LM, Santorelli FM (2009). Friedreich’s ataxia: oxidative stress and cytoskeletal abnormalities. *The Journal of the Neurological Sciences*.

[88] Santos R, Lefevre S, Sliwa D, Seguin A, Camadro J, Lesuisse E (2010). Friedreich ataxia: molecular mechanisms, redox considerations, and therapeutic opportunities. *Antioxidants and Redox Signaling*.

[89] Hyson HC, Kieburtz K, Shoulson I (2010). Safety and tolerability of high-dosage coenzyme Q(10) in Huntington’s disease and healthy subjects. *Movement Disorders*.

[90] Valencia A, Sapp E, Kimm JS (2013). Elevated NADPH oxidase activity contributes to oxidative stress and cell death in Huntington's disease. *Human Molecular Genetics*.

[91] Yang L, Calingasan NY, Wille EJ (2009). Combination therapy with Coenzyme Q(10) and creatine produces additive neuroprotective effects in models of Parkinson’s and Huntington’s diseases. *Journal of Neurochemistry*.

[92] Ribeiro M, Silva AC, Rodrigues J (2013). Oxidizing effects of exogenous stressors in Huntington's disease knock-in striatal cells—protective effect of cystamine and creatine. *Toxicological Sciences*.

[93] Tyagi N, Ovechkin AV, Lominadze D, Moshal KS, Tyagi SC (2006). Mitochondrial mechanism of microvascular endothelial cells apoptosis in hyperhomocysteinemia. *Journal of Cellular Biochemistry*.

[94] Moshal KS, Tipparaju SM, Vacek TP (2008). Mitochondrial matrix metalloproteinase activation decreases myocyte contractility in hyperhomocysteinemia. *The American Journal of Physiology—Heart and Circulatory Physiology*.

[95] Vanzin CS, Biancini GB, Sitta A (2011). Experimental evidence of oxidative stress in plasma of homocystinuric patients: a possible role for homocysteine. *Molecular Genetics and Metabolism*.

[96] Nur E, Brandjes DP, Schnog JB (2010). Plasma levels of advanced glycation end products are associated with haemolysis-related organ complications in sickle cell patients. *British Journal of Haematology*.

[97] Amer J, Ghoti H, Rachmilewitz E, Koren A, Levin C, Fibach E (2006). Red blood cells, platelets and polymorphonuclear neutrophils of patients with sickle cell disease exhibit oxidative stress that can be ameliorated by antioxidants. *British Journal of Haematology*.

[98] Grasso JA, Sullivan AL, Sullivan LW (1975). Ultrastructural studies of the bone marrow in sickle cell anaemia. I. The structure of sickled erythrocytes and reticulocytes and their phagocytic destruction. *British Journal of Haematology*.

[99] Hershko C (2010). Pathogenesis and management of iron toxicity in thalassemia. *Annals of the New York Academy of Sciences*.

[100] Ferro E, Visalli G, Civa R (2012). Oxidative damage and genotoxicity biomarkers in transfused and untransfused thalassemic subjects. *Free Radical Biology and Medicine*.

[101] Tsagris V, Liapi-Adamidou G (2005). Serum carnitine levels in patients with homozygous beta thalassemia: a possible new role for carnitine?. *European Journal of Pediatrics*.

[102] Rebrin I, Sohal RS (2008). Pro-oxidant shift in glutathione redox state during aging. *Advanced Drug Delivery Reviews*.

[103] Møller P, Løhr M, Folkmann JK, Mikkelsen L, Loft S (2010). Aging and oxidatively damaged nuclear DNA in animal organs. *Free Radical Biology and Medicine*.

[104] Cau SB, Carneiro FS, Tostes RC (2012). Differential modulation of nitric oxide synthases in aging: therapeutic opportunities. *Frontiers in Physiology*.

[105] Musicco C, Capelli V, Pesce V (2009). Accumulation of overoxidized Peroxiredoxin III in aged rat liver mitochondria. *Biochimica et Biophysica Acta—Bioenergetics*.

[106] Wang CH, Wu SB, Wu YT (2013). Oxidative stress response elicited by mitochondrial dysfunction: implication in the pathophysiology of aging. *Experimental Biology and Medicine*.

[107] Gomez-Cabrera MC, Sanchis-Gomar F, Garcia-Valles R (2012). Mitochondria as sources and targets of damage in cellular aging. *Clinical Chemistry and Laboratory Medicine*.

[108] Takimoto E, Kass DA (2007). Role of oxidative stress in cardiac hypertrophy and remodeling. *Hypertension*.

[109] Victor VM, Rocha M, Solá E, Bañuls C, Garcia-Malpartida K, Hernández-Mijares A (2009). Oxidative stress, endothelial dysfunction and atherosclerosis. *Current Pharmaceutical Design*.

[110] Di Lisa F, Kaludercic N, Carpi A, Menabò R, Giorgio M (2009). Mitochondria and vascular pathology. *Pharmacological Reports*.

[111] Perrotta I, Perrotta E, Sesti S (2013). MnSOD expression in human atherosclerotic plaques: an immunohistochemical and ultrastructural study. *Cardiovascular Pathology*.

[112] Sobenin IA, Sazonova MA, Postnov AY (2013). Changes of mitochondria in atherosclerosis: possible determinant in the pathogenesis of the disease. *Atherosclerosis*.

[113] Guzik B, Sagan A, Ludew D (2013). Mechanisms of oxidative stress in human aortic aneurysms—association with clinical risk factors for atherosclerosis and disease severity. *International Journal of Cardiology*.

[114] Hansel B, Giral P, Nobecourt E (2004). Metabolic syndrome is associated with elevated oxidative stress and dysfunctional dense high-density lipoprotein particles displaying impaired antioxidative activity. *The Journal of Clinical Endocrinology and Metabolism*.

[115] Palmieri VO, Grattagliano I, Portincasa P, Palasciano G (2006). Systemic oxidative alterations are associated with visceral adiposity and liver steatosis in patients with metabolic syndrome. *The Journal of Nutrition*.

[116] Koene S, Rodenburg RJ, van der Knaap MS (2012). Natural disease course and genotype-phenotype correlations in Complex I deficiency caused by nuclear gene defects: what we learned from 130 cases. *Journal of Inherited Metabolic Diseases*.

[117] Huang C, Su S, Hsieh M (2011). Depleted leukocyte mitochondrial DNA copy number in metabolic syndrome. *Journal of Atherosclerosis and Thrombosis*.

[118] Mitchell T, Darley-Usmar V (2012). Metabolic syndrome and mitochondrial dysfunction: insights from preclinical studies with a mitochondrially targeted antioxidant. *Free Radical Biology and Medicine*.

[119] Auinger A, Rubin D, Sabandal M (2013). A common haplotype of carnitine palmitoyltransferase 1b is associated with the metabolic syndrome. *British Journal of Nutrition*.

[120] Altindag O, Erel O, Aksoy N, Selek S, Celik H, Karaoglanoglu M (2007). Increased oxidative stress and its relation with collagen metabolism in knee osteoarthritis. *Rheumatology International*.

[121] Fernandez-Moreno M, Soto-Hermida A, Pertega S (2011). Mitochondrial DNA (mtDNA) haplogroups and serum levels of anti-oxidant enzymes in patients with osteoarthritis. *BMC Musculoskeletal Disorders*.

[122] Li Z, Shen J, Chen Y (2012). Mitochondrial genome sequencing of chondrocytes in osteoarthritis by human mitochondria RT2 Profiler PCR array. *Molecular Medicine Reports*.

[123] Fernández-Moreno M, Soto-Hermida A, Oreiro N (2012). Mitochondrial haplogroups define two phenotypes of osteoarthritis. *Frontiers in Physiology*.

[124] Gavrilidis C, Miwa S, von Zglinicki T (2013). Mitochondrial dysfunction in osteoarthritis is associated with down-regulation of superoxide dismutase 2. *Arthritis and Rheumatism*.

[125] Tabak O, Gelisgen R, Erman H (2011). Oxidative lipid, protein, and DNA damage as oxidative stress markers in vascular complications of diabetes mellitus. *Clinical and Investigative Medicine*.

[126] Remor AP, de Matos FJ, Ghisoni K (2011). Differential effects of insulin on peripheral diabetes-related changes in mitochondrial bioenergetics: involvement of advanced glycosylated end products. *Biochimica et Biophysica Acta—Molecular Basis of Disease*.

[127] Bansal S, Siddarth M, Chawla D, Banerjee BD, Madhu SV, Tripathi AK (2012). Advanced glycation end products enhance reactive oxygen and nitrogen species generation in neutrophils in vitro. *Molecular and Cellular Biochemistry*.

[128] Noriega-Cisneros R, Cortés-Rojo C, Manzo-Avalos S (2013). Mitochondrial response to oxidative and nitrosative stress in early stages of diabetes. *Mitochondrion*.

[129] Amer MA, Ghattas MH, Abo-Elmatty DM (2011). Influence of glutathione S-transferase polymorphisms on type-2 diabetes mellitus risk. *Genetics and Molecular Research*.

[130] Khan S, Raghuram GV, Bhargava A (2011). Role and clinical significance of lymphocyte mitochondrial dysfunction in type 2 diabetes mellitus. *Translational Research*.

[131] Avila C, Huang RJ, Stevens MV (2012). Platelet mitochondrial dysfunction is evident in type 2 diabetes in association with modifications of mitochondrial anti-oxidant stress proteins. *Experimental and Clinical Endocrinology and Diabetes*.

[132] Calabrese V, Cornelius C, Leso V (2012). Oxidative stress, glutathione status, sirtuin and cellular stress response in type 2 diabetes. *Biochimica et Biophysica Acta—Molecular Basis of Disease*.

[133] Gray SP, Di Marco E, Okabe J (2013). NADPH oxidase 1 plays a key role in diabetes mellitus-accelerated atherosclerosis. *Circulation*.

[134] Area-Gomez E, Del Carmen Lara Castillo M, Tambini MD (2012). Upregulated function of mitochondria-associated ER membranes in Alzheimer disease. *The EMBO Journal*.

[135] Atamna H, Frey WH (2007). Mechanisms of mitochondrial dysfunction and energy deficiency in Alzheimer’s disease. *Mitochondrion*.

[136] Dumont M, Kipiani K, Yu F (2011). Coenzyme Q(10) decreases amyloid pathology and improves behavior in a transgenic mouse model of alzheimer’s disease. *The Journal of Alzheimer’s Disease*.

[137] Murakami K, Murata N, Noda Y (2012). Stimulation of the amyloidogenic pathway by cytoplasmic superoxide radicals in an Alzheimer's disease mouse model. *Bioscience, Biotechnology, and Biochemistry*.

[138] Hardas SS, Sultana R, Clark AM (2013). Oxidative modification of lipoic acid by HNE in Alzheimer disease brain. *Redox Biology*.

[139] Aliev G, Palacios HH, Gasimov E (2010). Oxidative stress induced mitochondrial failure and vascular hypoperfusion as a key initiator for the development of alzheimer disease. *Pharmaceuticals*.

[140] Zarrouk A, Vejux A, Nury T (2012). Induction of mitochondrial changes associated with oxidative stress on very long chain fatty acids (C22:0, C24:0, or C26:0)-treated human neuronal cells (SK-NB-E). *Oxidative Medicine and Cellular Longevity*.

[141] Kou J, Kovacs GG, Höftberger R (2011). Peroxisomal alterations in Alzheimer's disease. *Acta Neuropathologica*.

[142] Cozzolino M, Carrì MT (2012). Mitochondrial dysfunction in ALS. *Progress in Neurobiology*.

[143] Cova E, Bongioanni P, Cereda C (2010). Time course of oxidant markers and antioxidant defenses in subgroups of amyotrophic lateral sclerosis patients. *Neurochemistry International*.

[144] Karch CM, Prudencio M, Winkler DD, Hart PJ, Borchelt DR (2009). Role of mutant SOD1 disulfide oxidation and aggregation in the pathogenesis of familial ALS. *Proceedings of the National Academy of Sciences of the United States of America*.

[145] Rodríguez GE, González DM, Monachelli GM (2012). Morphological abnormalities in mitochondria of the skin of patients with sporadic amyotrophic lateral sclerosis. *Arquivos de Neuro-Psiquiatria*.

[146] Ghiasi P, Hosseinkhani S, Noori A, Nafissi S, Khajeh K (2012). Mitochondrial complex I deficiency and ATP/ADP ratio in lymphocytes of amyotrophic lateral sclerosis patients. *Neurological Research*.

[147] Chuang Y (2010). Mitochondrial dysfunction and oxidative stress in seizure-induced neuronal cell death. *Acta Neurologica Taiwanica*.

[148] Malinska D, Kulawiak B, Kudin AP (2010). Complex III-dependent superoxide production of brain mitochondria contributes to seizure-related ROS formation. *Biochimica et Biophysica Acta—Bioenergetics*.

[149] Mayr JA, Zimmermann FA, Fauth C (2011). Lipoic acid synthetase deficiency causes neonatal-onset epilepsy, defective mitochondrial energy metabolism, and glycine elevation. *American Journal of Human Genetics*.

[150] García-Giménez JL, Seco-Cervera M, Aguado C (2013). Lafora disease fibroblasts exemplify the molecular interdependence between thioredoxin 1 and the proteasome in mammalian cells. *Free Radical Biology and Medicine C*.

[151] Booth NE, Myhill S, McLaren-Howard J (2012). Mitochondrial dysfunction and the pathophysiology of Myalgic Encephalomyelitis/Chronic Fatigue Syndrome (ME/CFS). *International Journal of Clinical and Experimental Medicine*.

[152] Maes M, Mihaylova I, Kubera M, Uytterhoeven M, Vrydags N, Bosmans E (2009). Increased 8-hydroxy-deoxyguanosine, a marker of oxidative damage to DNA, in major depression and myalgic encephalomyelitis/chronic fatigue syndrome. *Neuroendocrinology Letters*.

[153] Maes M, Mihaylova I, Kubera M, Uytterhoeven M, Vrydags N, Bosmans E (2009). Coenzyme Q(10) deficiency in myalgic encephalomyelitis/chronic fatigue syndrome (ME/CFS) is related to fatigue, autonomic and neurocognitive symptoms and is another risk factor explaining the early mortality in ME/CFS due to cardiovascular disorder. *Neuroendocrinology Letters*.

[154] Maes M, Kubera M, Uytterhoeven M, Vrydags N, Bosmans E (2011). Increased plasma peroxides as a marker of oxidative stress in myalgic encephalomyelitis/chronic fatigue syndrome (ME/CFS). *Medical Science Monitor*.

[155] Jammes Y, Steinberg JG, Delliaux S (2012). Chronic fatigue syndrome: acute infection and history of physical activity affect resting levels and response to exercise of plasma oxidant/antioxidant status and heat shock proteins. *Journal of Internal Medicine*.

[156] Smits B, van den Heuvel L, Knoop H (2011). Mitochondrial enzymes discriminate between mitochondrial disorders and chronic fatigue syndrome. *Mitochondrion*.

[157] Miller E, Walczak A, Saluk J, Ponczek MB, Majsterek I (2012). Oxidative modification of patient’s plasma proteins and its role in pathogenesis of multiple sclerosis. *Clinical Biochemistry*.

[158] Fischer MT, Sharma R, Lim JL (2012). NADPH oxidase expression in active multiple sclerosis lesions in relation to oxidative tissue damage and mitochondrial injury. *Brain*.

[159] Campbell GR, Mahad DJ (2012). Clonal expansion of mitochondrial DNA deletions and the progression of multiple sclerosis. *CNS and Neurological Disorders—Drug Targets*.

[160] Miller E, Walczak A, Majsterek I (2013). Melatonin reduces oxidative stress in the erythrocytes of multiple sclerosis patients with secondary progressive clinical course. *Journal of Neuroimmunology*.

[161] Seet RCS, Lee CJ, Lim ECH (2010). Oxidative damage in Parkinson disease: measurement using accurate biomarkers. *Free Radical Biology and Medicine*.

[162] Mischley LK, Allen J, Bradley R (2012). Coenzyme Q(10) deficiency in patients with Parkinson’s disease. *The Journal of the Neurological Sciences*.

[163] Naoi M, Maruyama W, Shamoto-Nagai M, Yi H, Akao Y, Tanaka M (2005). Oxidative stress in mitochondria: decision to survival and death of neurons in neurodegenerative disorders. *Molecular Neurobiology*.

[164] Danielson SR, Held JM, Oo M, Riley R, Gibson BW, Andersen JK (2011). Quantitative mapping of reversible mitochondrial complex I cysteine oxidation in a Parkinson disease mouse model. *The Journal of Biological Chemistry*.

[165] Cacciatore I, Baldassarre L, Fornasari E (2012). Recent advances in the treatment of neurodegenerative diseases based on GSH delivery systems. *Oxidative Medicine and Cellular Longevity*.

[166] Rose S, Melnyk S, Trusty TA (2012). Intracellular and extracellular redox status and free radical generation in primary immune cells from children with autism. *Autism Research and Treatment*.

[167] Pecorelli A, Leoncini S, de Felice C (2013). Non-protein-bound iron and 4-hydroxynonenal protein adducts in classic autism. *Brain and Development*.

[168] Frustaci A, Neri M, Cesario A (2012). Oxidative stress-related biomarkers in autism: systematic review and meta-analyses. *Free Radical Biology and Medicine*.

[169] Rose S, Melnyk S, Pavliv O (2012). Evidence of oxidative damage and inflammation associated with low glutathione redox status in the autism brain. *Translational Psychiatry*.

[170] Anitha A, Nakamura K, Thanseem I (2013). Downregulation of the expression of mitochondrial electron transport complex genes in autism brains. *Brain Pathology*.

[171] Napoli E, Wong S, Giulivi C (2013). Evidence of reactive oxygen species-mediated damage to mitochondrial DNA in children with typical autism. *Molecular Autism*.

[172] Ross-Inta C, Omanska-Klusek A, Wong S (2010). Evidence of mitochondrial dysfunction in fragile X-associated tremor/ataxia syndrome. *Biochemical Journal*.

[173] Banerjee U, Dasgupta A, Rout JK, Singh OP (2012). Effects of lithium therapy on Na^+^-K^+^-ATPase activity and lipid peroxidation in bipolar disorder. *Progress in Neuro-Psychopharmacology and Biological Psychiatry*.

[174] Raffa M, Barhoumi S, Atig F (2012). Reduced antioxidant defense systems in schizophrenia and bipolar I disorder. *Progress in Neuro-Psychopharmacology and Biological Psychiatry*.

[175] Andreazza AC, Wang JF, Salmasi F (2013). Specific subcellular changes in oxidative stress in prefrontal cortex from patients with bipolar disorder. *Journal of Neurochemistry*.

[176] Rawdin BS, Mellon SH, Dhabhar FS (2013). Dysregulated relationship of inflammation and oxidative stress in major depression. *Brain, Behavior, and Immunity*.

[177] Szuster-Ciesielska A, Słotwińska M, Stachura A (2008). Accelerated apoptosis of blood leukocytes and oxidative stress in blood of patients with major depression. *Progress in Neuro-Psychopharmacology and Biological Psychiatry*.

[178] Gibson SA, Korade Ž, Shelton RC (2012). Oxidative stress and glutathione response in tissue cultures from persons with major depression. *Journal of Psychiatric Research*.

[179] Ben-Shachar D, Karry R (2008). Neuroanatomical pattern of mithochondrial complex I pathology varies between schizoprenia, bipolar disorder and major depression. *PLoS ONE*.

[180] Chakraborty S, Singh OP, Dasgupta A, Mandal N, Das HN (2009). Correlation between lipid peroxidation-induced TBARS level and disease severity in obsessive-compulsive disorder. *Progress in Neuro-Psychopharmacology and Biological Psychiatry*.

[181] Behl A, Swami G, Sircar SS, Bhatia MS, Banerjee BD (2010). Relationship of possible stress-related biochemical markers to oxidative/antioxidative status in obsessive-compulsive disorder. *Neuropsychobiology*.

[182] Orhan N, Kucukali CI, Cakir U, Seker N, Aydin M (2012). Genetic variants in nuclear-encoded mitochondrial proteins are associated with oxidative stress in obsessive compulsive disorders. *Journal of Psychiatric Research*.

[183] Anderson G, Maes M, Berk M (2013). Schizophrenia is primed for an increased expression of depression through activation of immuno-inflammatory, oxidative and nitrosative stress, and tryptophan catabolite pathways. *Progress in Neuro-Psychopharmacology and Biological Psychiatry*.

[184] Pedrini M, Massuda R, Fries GR (2012). Similarities in serum oxidative stress markers and inflammatory cytokines in patients with overt schizophrenia at early and late stages of chronicity. *Journal of Psychiatric Research*.

[185] Kulak A, Steullet P, Cabungcal JH (2012). Redox dysregulation in the pathophysiology of schizophrenia and bipolar disorder: insights from animal models. *Antioxidants and Redox Signaling*.

[186] Zhang XY, Chen DC, Xiu MH (2012). Plasma total antioxidant status and cognitive impairments in schizophrenia. *Schizophrenia Research*.

[187] Gubert C, Stertz L, Pfaffenseller B (2013). Mitochondrial activity and oxidative stress markers in peripheral blood mononuclear cells of patients with bipolar disorder, schizophrenia, and healthy subjects. *Journal of Psychiatric Research*.

[188] Costa A, Scholer-Dahirel A, Mechta-Grigoriou F (2014). The role of reactive oxygen species and metabolism on cancer cells and their microenvironment. *Seminars in Cancer Biology*.

[189] Hanahan D, Weinberg RA (2011). Hallmarks of cancer: the next generation. *Cell*.

[190] Masoudi-Nejad A, Asgari Y (2014). Metabolic Cancer Biology: structural-based analysis of cancer as a metabolic disease, new sights and opportunities for disease treatment. *Seminars in Cancer Biology*.

[191] Soini Y, Haapasaari K, Vaarala MH, Turpeenniemi-Hujanen T, Kärjä V, Karihtala P (2011). 8-hydroxydeguanosine and nitrotyrosine are prognostic factors in urinary bladder carcinoma. *International Journal of Clinical and Experimental Pathology*.

[192] Badjatia N, Satyam A, Singh P, Seth A, Sharma A (2010). Altered antioxidant status and lipid peroxidation in Indian patients with urothelial bladder carcinoma. *Urologic Oncology: Seminars and Original Investigations*.

[193] Guney AI, Ergec DS, Tavukcu HH (2012). Detection of mitochondrial DNA mutations in nonmuscle invasive bladder cancer. *Genetic Testing and Molecular Biomarkers*.

[194] Niu HT, Yang CM, Jiang G (2011). Cancer stroma proteome expression profile of superficial bladder transitional cell carcinoma and biomarker discovery. *The Journal of Cancer Research and Clinical Oncology*.

[195] Kedzierska M, Olas B, Wachowicz B, Jeziorski A, Piekarski J (2012). Relationship between thiol, tyrosine nitration and carbonyl formation as biomarkers of oxidative stress and changes of hemostatic function of plasma from breast cancer patients before surgery. *Clinical Biochemistry*.

[196] Pande D, Negi R, Karki K (2012). Oxidative damage markers as possible discriminatory biomarkers in breast carcinoma. *Translational Research*.

[197] Martinez-Outschoorn UE, Balliet R, Lin Z (2012). BRCA1 mutations drive oxidative stress and glycolysis in the tumor microenvironment: implications for breast cancer prevention with antioxidant therapies. *Cell Cycle*.

[198] Salem AF, Howell A, Sartini M (2012). Downregulation of stromal BRCA1 drives breast cancer tumor growth via upregulation of HIF-1*α*, autophagy and ketone body production. *Cell Cycle*.

[199] Sotgia F, Whitaker-Menezes D, Martinez-Outschoorn UE (2012). Mitochondria, “fuel” breast cancer metabolism: fifteen markers of mitochondrial biogenesis label epithelial cancer cells, but are excluded from adjacent stromal cell. *Cell Cycle*.

[200] Coene ED, Hollinshead MS, Waeytens AAT (2005). Phosphorylated BRCA1 is predominantly located in the nucleus and mitochondria. *Molecular Biology of the Cell*.

[201] Cooney RV, Dai Q, Gao Y (2011). Low plasma coenzyme Q(10) levels and breast cancer risk in Chinese women. *Cancer Epidemiology Biomarkers and Prevention*.

[202] Looi M, Ahmad Zailani Hatta AZH, Dali AZHM, Ali SAM, Ngah WZW, Yusof YAM (2008). Oxidative damage and antioxidant status in patients with cervical intraepithelial neoplasia and carcinoma of the cervix. *European Journal of Cancer Prevention*.

[203] Li L, Chen C, Cao Z (2009). Expression of peroxiredoxin III cervical lesions. *ZZhonghua Shi Yan He Lin Chuang Bing Du Xue Za Zhi*.

[204] Srivastava S, Natu SM, Gupta A (2009). Lipid peroxidation and antioxidants in different stages of cervical cancer: prognostic significance. *Indian Journal of Cancer*.

[205] Sharma A, Rajappa M, Satyam A, Sharma M (2010). Oxidant/anti-oxidant dynamics in patients with advanced cervical cancer: correlation with treatment response. *Molecular and Cellular Biochemistry*.

[206] de Marco F, Bucaj E, Foppoli C (2012). Oxidative stress in HPV-driven viral carcinogenesis: redox proteomics analysis of HPV-16 dysplastic and neoplastic tissues. *PLoS ONE*.

[207] Gao L, Pan X, Li L (2011). Null genotypes of gstm1 and gstt1 contribute to risk of cervical neoplasia: an evidence-based meta-analysis. *PLoS ONE*.

[208] Safaeian M, Hildesheim A, Gonzalez P (2012). Single nucleotide polymorphisms in the PRDX3 and RPS19 and risk of HPV persistence and cervical precancer/cancer. *PLoS ONE*.

[209] Strzelczyk JK, Wielkoszyński T, Krakowczyk Ł (2012). The activity of antioxidant enzymes in colorectal adenocarcinoma and corresponding normal mucosa. *Acta Biochimica Polonica*.

[210] Scarpa M, Cardin R, Bortolami M (2013). Mucosal immune environment in colonic carcinogenesis: CD80 expression is associated to oxidative DNA damage and TLR4-NF*κ*B signalling. *European Journal of Cancer*.

[211] Thyagarajan B, Wang R, Barcelo H (2012). Mitochondrial copy number is associated with colorectal cancer risk. *Cancer Epidemiology, Biomarkers and Prevention*.

[212] Pejić S, Todorović A, Stojiljković V (2009). Antioxidant enzymes and lipid peroxidation in endometrium of patients with polyps, myoma, hyperplasia and adenocarcinoma. *Reproductive Biology and Endocrinology*.

[213] Cormio A, Guerra F, Cormio G (2009). The PGC-1*α*-dependent pathway of mitochondrial biogenesis is upregulated in type I endometrial cancer. *Biochemical and Biophysical Research Communications*.

[214] Guerra F, Kurelac I, Cormio A (2011). Placing mitochondrial DNA mutations within the progression model of type I endometrial carcinoma. *Human Molecular Genetics*.

[215] Ni J, Mei M, Sun L (2012). Oxidative DNA damage and repair in chronic atrophic gastritis and gastric cancer. *Hepato-Gastroenterology*.

[216] Uehara T, Ma D, Yao Y (2013). *H. pylori* infection is associated with DNA damage of Lgr5-positive epithelial stem cells in the stomach of patients with gastric cancer. *Digestive Diseases and Sciences*.

[217] Machado AM, Desler C, Bøggild S (2013). *Helicobacter pylori* infection affects mitochondrial function and DNA repair, thus, mediating genetic instability in gastric cells. *Mechanisms of Ageing and Development*.

[218] Suzuki Y, Imai K, Takai K (2013). Hepatocellular carcinoma patients with increased oxidative stress levels are prone to recurrence after curative treatment: a prospective case series study using the d-ROM test. *The Journal of Cancer Research and Clinical Oncology*.

[219] Tanaka S, Miyanishi K, Kobune M (2013). Increased hepatic oxidative DNA damage in patients with nonalcoholic steatohepatitis who develop hepatocellular carcinoma. *Journal of Gastroenterology*.

[220] Tamai T, Uto H, Takami Y (2011). Serum manganese superoxide dismutase and thioredoxin are potential prognostic markers for hepatitis C virus-related hepatocellular carcinoma. *World Journal of Gastroenterology*.

[221] Jin Y, Yu Q, Zhou D (2012). The mitochondrial DNA 9-bp deletion polymorphism is a risk factor for hepatocellular carcinoma in the Chinese population. *Genetic Testing and Molecular Biomarkers*.

[222] Duan W, Hua R, Yi W (2012). The association between OGG1 Ser326Cys polymorphism and lung cancer susceptibility: a meta-analysis of 27 studies. *PLoS ONE*.

[223] Cobanoglu U, Demir H, Cebi A (2011). Lipid peroxidation, DNA damage and coenzyme Q(10) in lung cancer patients—markers for risk assessment?. *Asian Pacific Journal of Cancer Prevention*.

[224] Janik J, Swoboda M, Janowska B (2011). 8-Oxoguanine incision activity is impaired in lung tissues of NSCLC patients with the polymorphism of OGG1 and XRCC1 genes. *Mutation Research—Fundamental and Molecular Mechanisms of Mutagenesis*.

[225] Tsai JR, Wang HM, Liu PL (2012). High expression of heme oxygenase-1 is associated with tumor invasiveness and poor clinical outcome in non-small cell lung cancer patients. *Cell Oncology*.

[226] Ito K, Yano T, Morodomi Y (2012). Serum antioxidant capacity and oxidative injury to pulmonary DNA in never-smokers with primary lung cancer. *Anticancer Research*.

[227] Ye X, Li Q, Wang G (2011). Mitochondrial and energy metabolism-related properties as novel indicators of lung cancer stem cells. *International Journal of Cancer*.

[228] Chae YC, Caino MC, Lisanti S (2012). Control of tumor bioenergetics and survival stress signaling by mitochondrial HSP90s. *Cancer Cell*.

[229] Murtas D, Piras F, Minerba L (2010). Nuclear 8-hydroxy-2′-deoxyguanosine as survival biomarker in patients with cutaneous melanoma. *Oncology Reports*.

[230] Ibarrola-Villava M, Peña-Chilet M, Fernandez LP (2011). Genetic polymorphisms in DNA repair and oxidative stress pathways associated with malignant melanoma susceptibility. *European Journal of Cancer*.

[231] Lin X, Zheng W, Liu J (2013). Oxidative stress in malignant melanoma enhances Tumor Necrosis Factor-*α* secretion of tumor-associated macrophages that promote cancer cell invasion. *Antioxidants and Redox Signaling*.

[232] Barbi de Moura M, Vincent G, Fayewicz SL (2012). Mitochondrial respiration—an important therapeutic target in melanoma. *PLoS ONE*.

[233] Singh RK, Tripathi AK, Tripathi P, Singh S, Singh R, Ahmad R (2009). Studies on biomarkers for oxidative stress in patients with chronic myeloid leukemia. *Hematology/Oncology and Stem Cell Therapy*.

[234] Sailaja K, Surekha D, Nageswara Rao D, Raghunadha Rao D, Vishnupriya S (2010). Association of the GSTP1 gene (Ile105Val) Polymorphism with chronic myeloid leukemia. *Asian Pacific Journal of Cancer Prevention*.

[235] Ahmad R, Tripathi AK, Tripathi P, Singh R, Singh S, Singh RK (2010). Studies on lipid peroxidation and non-enzymatic antioxidant status as indices of oxidative stress in patients with chronic myeloid leukaemia. *Singapore Medical Journal*.

[236] Zhou F, Zhang W, Wei Y (2010). Involvement of oxidative stress in the relapse of acute myeloid leukemia. *The Journal of Biological Chemistry*.

[237] Palande KK, Beekman R, van der Meeren LE, Berna Beverloo H, Valk PJM, Touw IP (2011). The antioxidant protein peroxiredoxin 4 is epigenetically down regulated in acute promyelocytic leukemia. *PLoS ONE*.

[238] Akinlolu A, Akingbola T, Salau B (2012). Lipid peroxidation in Nigerians affected with haematological malignancies. *African Journal of Medicine and Medical Sciences*.

[239] Boultwood J, Fidler C, Mills KI (1996). Amplification of mitochondrial DNA in acute myeloid leukaemia. *British Journal of Haematology*.

[240] Koistinen P, Ruuska S, Säily M (2006). An association between manganese superoxide dismutase polymorphism and outcome of chemotherapy in acute myeloid leukemia. *Haematologica*.

[241] Schildgen V, Wulfert M, Gattermann N (2011). Impaired mitochondrial gene transcription in myelodysplastic syndromes and acute myeloid leukemia with myelodysplasia-related changes. *Experimental Hematology*.

[242] Gokul S, Patil VS, Jailkhani R, Hallikeri K, Kattappagari KK (2010). Oxidant-antioxidant status in blood and tumor tissue of oral squamous cell carcinoma patients. *Oral Diseases*.

[243] Korde SD, Basak A, Chaudhary M, Goyal M, Vagga A (2011). Enhanced nitrosative and oxidative stress with decreased total antioxidant capacity in patients with oral precancer and oral squamous cell carcinoma. *Oncology*.

[244] Marakala V, Malathi M, Shivashankara AR (2012). Lipid peroxidation and antioxidant vitamin status in oral cavity and oropharyngeal cancer patients. *Asian Pacific Organization for Cancer Prevention*.

[245] Vlková B, Stanko P, Minárik G (2012). Salivary markers of oxidative stress in patients with oral premalignant lesions. *Archives of Oral Biology*.

[246] Liu S, Jiang R, Chen F, Wang W, Lin J (2012). Somatic mutations in the D-loop of mitochondrial DNA in oral squamous cell carcinoma. *European Archives of Oto-Rhino-Laryngology*.

[247] Gasparre G, Porcelli AM, Bonora E (2007). Disruptive mitochondrial DNA mutations in complex I subunits are markers of oncocytic phenotype in thyroid tumors. *Proceedings of the National Academy of Sciences of the United States of America*.

[248] Bonora E, Porcelli AM, Gasparre G (2006). Defective oxidative phosphorylation in thyroid oncocytic carcinoma is associated with pathogenic mitochondrial DNA mutations affecting complexes I and III. *Cancer Research*.

[249] Sciascia S, Roccatello D, Bertero MT (2012). 8-isoprostane, prostaglandin E2, C-reactive protein and serum amyloid A as markers of inflammation and oxidative stress in antiphospholipid syndrome: a pilot study. *Inflammation Research*.

[250] Perez-Sanchez C, Ruiz-Limon P, Aguirre MA (2012). Mitochondrial dysfunction in antiphospholipid syndrome: implications in the pathogenesis of the disease and effects of coenzyme Q(10) treatment. *Blood*.

[251] Naziroğlu M, Kökçam I, Simşek H (2003). Lipid peroxidation and antioxidants in plasma and red blood cells from patients with pemphigus vulgaris. *Journal of Basic and Cinical Physiology and Pharmacology*.

[252] Abida O, Ben Mansour R, Gargouri B (2012). Catalase and lipid peroxidation values in serum of Tunisian patients with pemphigus vulgaris and foliaceus. *Biological Trace Element Research*.

[253] Marchenko S, Chernyavsky AI, Arredondo J, Gindi V, Grando SA (2010). Antimitochondrial autoantibodies in pemphigus vulgaris: a missing link in disease pathophysiology. *The Journal of Biological Chemistry*.

[254] Kalantari-Dehaghi M, Chen Y, Deng W (2013). Mechanisms of mitochondrial damage in keratinocytes by pemphigus vulgaris antibodies. *The Journal of Biological Chemistry*.

[255] Cash WJ, McCance DR, Young IS (2010). Primary biliary cirrhosis is associated with oxidative stress and endothelial dysfunction but not increased cardiovascular risk. *Hepatology Research*.

[256] Sorrentino P, Terracciano L, D’Angelo S (2010). Oxidative stress and steatosis are cofactors of liver injury in primary biliary cirrhosis. *Journal of Gastroenterology*.

[257] Salunga TL, Cui Z, Shimoda S (2007). Oxidative stress-induced apoptosis of bile duct cells in primary biliary cirrhosis. *Journal of Autoimmunity*.

[258] Leung PSC, Rossaro L, Davis PA (2007). Antimitochondrial antibodies in acute liver failure: implications for primary biliary cirrhosis. *Hepatology*.

[259] Basavaraj KH, Vasu Devaraju P, Rao KS (2013). Studies on serum 8-hydroxy guanosine (8-OHdG) as reliable biomarker for psoriasis. *Journal of the European Academy of Dermatology and Venereology*.

[260] Kaur S, Zilmer K, Leping V (2013). Serum methylglyoxal level and its association with oxidative stress and disease severity in patients with psoriasis. *Archives of Dermatological Research*.

[261] Barygina VV, Becatti M, Soldi G (2013). Altered redox status in the blood of psoriatic patients: involvement of NADPH oxidase and role of anti-TNF-*α* therapy. *Redox Reports*.

[262] Gabr SA, Al-Ghadir AH (2012). Role of cellular oxidative stress and cytochrome c in the pathogenesis of psoriasis. *Archives of Dermatological Research*.

[263] Stark K, Törmä H, Oliw EH (2006). Co-localization of COX-2, CYP4F8, and mPGES-1 in epidermis with prominent expression of CYP4F8 mRNA in psoriatic lesions. *Prostaglandins and Other Lipid Mediators*.

[264] Shah D, Wanchu A, Bhatnagar A (2011). Interaction between oxidative stress and chemokines: possible pathogenic role in systemic lupus erythematosus and rheumatoid arthritis. *Immunobiology*.

[265] Connor AM, Mahomed N, Gandhi R, Keystone EC, Berger SA (2012). TNF*α* modulates protein degradation pathways in rheumatoid arthritis synovial fibroblasts. *Arthritis Research and Therapy*.

[266] Stamp LK, Khalilova I, Tarr JM (2012). Myeloperoxidase and oxidative stress in rheumatoid arthritis. *Rheumatology*.

[267] Kundu S, Ghosh P, Datta S (2012). Oxidative stress as a potential biomarker for determining disease activity in patients with rheumatoid arthritis. *Free Radical Research*.

[268] Ahmad S, Habib S, Moinuddin A (2013). Preferential recognition of epitopes on AGE-IgG by the autoantibodies in rheumatoid arthritis patients. *Human Immunology*.

[269] Harty LC, Biniecka M, O’Sullivan J (2012). Mitochondrial mutagenesis correlates with the local inflammatory environment in arthritis. *Annals of the Rheumatic Diseases*.

[270] Norheim KB, Jonsson G, Harboe E, Hanasand M, Gøransson L, Omdal R (2012). Oxidative stress, as measured by protein oxidation, is increased in primary Sjøgren’s syndrome. *Free Radical Research*.

[271] Cay HF, Sezer I, Dogan S (2012). Polymorphism in the TNF-*α* gene promoter at position −1031 is associated with increased circulating levels of TNF-*α*, myeloperoxidase and nitrotyrosine in primary Sjögren's Syndrome. *Clinical and Experimental Rheumatology*.

[272] Ryo K, Yamada H, Nakagawa Y (2007). Possible involvement of oxidative stress in salivary gland of patients with Sjögren’s syndrome. *Pathobiology*.

[273] Kurimoto C, Kawano S, Tsuji G (2007). Thioredoxin may exert a protective effect against tissue damage caused by oxidative stress in salivary glands of patients with Sjögren’s syndrome. *The Journal of Rheumatology*.

[274] Ikuno N, Mackay IR, Jois J, Omagari K, Rowley MJ (2001). Antimitochondrial autoantibodies in saliva and sera from patients with primary biliary cirrhosis. *Journal of Gastroenterology and Hepatology*.

[275] Fernandez D, Bonilla E, Phillips P, Perl A (2006). Signaling abnormalities in systemic lupus erythematosus as potential drug targets. *Endocrine, Metabolic and Immune Disorders—Drug Targets*.

[276] Perl A, Hanczko R, Doherty E (2012). Assessment of mitochondrial dysfunction in lymphocytes of patients with systemic lupus erythematosus. *Methods in Molecular Biology*.

[277] Hassan I, Sajad P, Majid S (2013). Serum antioxidant status in patients with systemic sclerosis. *Indian Journal of Dermatology*.

[278] Zalewska A, Knaś M, Gińdzieńska-Sieśkiewicz E (2013). Salivary antioxidants in patients with systemic sclerosis. *Journal of Oral Pathology and Medicine*.

[279] Cruz-Domínguez MP, Montes-Cortes DH, Olivares-Corichi IM (2013). Oxidative stress in Mexicans with diffuse cutaneous systemic sclerosis. *Rheumatology International*.

[280] Ohtsuka T (2008). Relation between elevated high-sensitivity C-reactive protein and anti-mitochondria antibody in patients with systemic sclerosis. *Journal of Dermatology*.

[281] Kostyuk VA, Potapovich AI, Cesareo E (2010). Dysfunction of glutathione S-transferase leads to excess 4-hydroxy-2-nonenal and H(2)O(2) and impaired cytokine pattern in cultured keratinocytes and blood of vitiligo patients. *Antioxidants and Redox Signaling*.

[282] Bassiouny DA, Khorshied MM (2013). Glutathione S-transferase M1 and T1 genetic polymorphism in Egyptian patients with nonsegmental vitiligo. *Clinical and Experimental Dermatology*.

[283] Dell’Anna ML, Ottaviani M, Bellei B (2010). Membrane lipid defects are responsible for the generation of reactive oxygen species in peripheral blood mononuclear cells from vitiligo patients. *Journal of Cellular Physiology*.

[284] Heydari B, Kazemi T, Zarban A (2012). Correlation of cataract with serum lipids, glucose and antioxidant activities: a case-control study. *West Indian Medical Journal*.

[285] Kisic B, Miric D, Zoric L, Ilic A, Dragojevic I (2012). Antioxidant capacity of lenses with age-related cataract. *Oxidative Medicine and Cellular Longevity*.

[286] Zhang Y, Zhang L, Sun D, Li Z, Wang L, Liu P (2011). Genetic polymorphisms of superoxide dismutases, catalase, and glutathione peroxidase in age-related cataract. *Molecular Vision*.

[287] Jarrett SG, Lewin AS, Boulton ME (2010). The importance of Mitochondria in age-related and inherited eye disorders. *Ophthalmic Research*.

[288] Lee W, Wells T, Kantorow M (2007). Localization and H(2)O(2)-specific induction of PRDX3 in the eye lens. *Molecular Vision*.

[289] Bagis S, Tamer L, Sahin G (2005). Free radicals and antioxidants in primary fibromyalgia: an oxidative stress disorder?. *Rheumatology International*.

[290] Altindag O, Celik H (2006). Total antioxidant capacity and the severity of the pain in patients with fibromyalgia. *Redox Report*.

[291] Cordero MD, Alcocer-Gómez E, Cano-García FJ (2011). Clinical symptoms in Fibromyalgia are better associated to lipid peroxidation levels in blood mononuclear cells rather than in plasma. *PLoS ONE*.

[292] Cordero MD, de Miguel M, Moreno Fernández AM (2010). Mitochondrial dysfunction and mitophagy activation in blood mononuclear cells of fibromyalgia patients: implications in the pathogenesis of the disease. *Arthritis Research and Therapy*.

[293] Cordero MD, Díaz-Parrado E, Carrión AM (2013). Is inflammation a mitochondrial dysfunction-dependent event in fibromyalgia?. *Antioxidants and Redox Signaling*.

[294] Običan S, Scialli AR (2011). Teratogenic exposures. *American Journal of Medical Genetics C: Seminars in Medical Genetics*.

[295] Wells PG, Bhuller Y, Chen CS (2005). Molecular and biochemical mechanisms in teratogenesis involving reactive oxygen species. *Toxicology and Applied Pharmacology*.

[296] Kovacic P, Somanathan R (2006). Mechanism of teratogenesis: electron transfer, reactive oxygen species, and antioxidants. *Birth Defects Research C—Embryo Today: Reviews*.

[297] Zheng X, Su M, Pei L (2011). Metabolic signature of pregnant women with neural tube defects in offspring. *Journal of Proteome Research*.

[298] Dong J, Sulik KK, Chen S (2010). The role of NOX enzymes in ethanol-induced oxidative stress and apoptosis in mouse embryos. *Toxicology Letters*.

[299] Fantel AG, Person RE (2002). Involvement of mitochondria and other free radical sources in normal and abnormal fetal development. *Annals of the New York Academy of Sciences*.

[300] Xu Y, Liu P, Li Y (2005). Impaired development of mitochondria plays a role in the central nervous system defects of fetal alcohol syndrome. *Birth Defects Research A—Clinical and Molecular Teratology*.

[301] Harman D (1956). Aging: a theory based on free radical and radiation chemistry. *The Journal of Gerontology*.

[302] Harman D (1972). Free radical theory of aging: dietary implications. *The American Journal of Clinical Nutrition*.

[303] Przyklenk K, Kloner RA (1987). Effect of oxygen-derived free radical scavengers on infarct size following six hours of permanent coronary artery occlusion: salvage or delay of myocyte necrosis?. *Basic Research in Cardiology*.

[304] Wolff SP, Bascal ZA, Hunt JV (1989). ‘Autoxidative glycosylation’: free radicals and glycation theory. *Progress in Clinical and Biological Research*.

[305] Clausen J (1984). Demential syndromes and the lipid metabolism. *Acta Neurologica Scandinavica*.

[306] Zemlan FP, Thienhaus OJ, Bosmann HB (1989). Superoxide dismutase activity in Alzheimer’s disease: possible mechanism for paired helical filament formation. *Brain Research*.

[307] Vives-Bauza C, Przedborski S (2011). Mitophagy: the latest problem for Parkinson’s disease. *Trends in Molecular Medicine*.

[308] Wołyniec E, Karpińska J, Losiewska S (2012). Determination of lipoic acid by flow-injection and high-performance liquid chromatography with chemiluminescence detection. *Talanta*.

[309] Pagano G, Castello G (2012). Oxidative stress and mitochondrial dysfunction in down syndrome. *Advances in Experimental Medicine and Biology*.

[310] Liu J (2008). The effects and mechanisms of mitochondrial nutrient alpha-lipoic acid on improving age-associated mitochondrial and cognitive dysfunction: an overview. *Neurochemical Research*.

[311] Tarnopolsky MA (2008). The mitochondrial cocktail: rationale for combined nutraceutical therapy in mitochondrial cytopathies. *Advanced Drug Delivery Reviews*.

[312] McMackin CJ, Widlansky ME, Hamburg NM (2007). Effect of combined treatment with alpha-Lipoic acid and acetyl-L-carnitine on vascular function and blood pressure in patients with coronary artery disease. *Journal of Clinical Hypertension*.

[313] Parikh S, Goldstein A, Koenig MK (2013). Practice patterns of mitochondrial disease physicians in North America, part 2: treatment, care and management. *Mitochondrion*.

[314] Pagano G, Aiello Talamanca A, Castello G Mitochondrial nutrients in disorders featuring oxidative stress and mitochondrial dysfunction: current experience and rational design of chemoprevention trials.

